# RhoA GEF Mcf2lb regulates rosette integrity during collective cell migration

**DOI:** 10.1242/dev.201898

**Published:** 2024-01-02

**Authors:** Hannah M. Olson, Amanda Maxfield, Nicholas L. Calistri, Laura M. Heiser, Weiyi Qian, Holger Knaut, Alex V. Nechiporuk

**Affiliations:** ^1^ Department of Cell, Developmental & Cancer Biology, Oregon Health & Science University, The Knight Cancer Institute, Portland, OR 97239, USA; ^2^ Neuroscience Graduate Program, Oregon Health & Science University, Portland, OR 97239, USA; ^3^ Biomedical Engineering, Oregon Health & Science University, Portland, OR 97239, USA; ^4^ Biomedical Engineering Graduate Program, Oregon Health & Science University, Portland, OR 97239, USA; ^5^ Skirball Institute of Biomolecular Medicine, New York University Grossman School of Medicine, New York, NY 10016, USA

**Keywords:** Cell migration, Rosette morphogenesis, Sensory development, Zebrafish lateral line, Apical constriction

## Abstract

Multicellular rosettes are transient epithelial structures that serve as important cellular intermediates in the formation of diverse organs. Using the zebrafish posterior lateral line primordium (pLLP) as a model system, we investigated the role of the RhoA GEF Mcf2lb in rosette morphogenesis. The pLLP is a group of ∼150 cells that migrates along the zebrafish trunk and is organized into epithelial rosettes; these are deposited along the trunk and will differentiate into sensory organs called neuromasts (NMs). Using single-cell RNA-sequencing and whole-mount *in situ* hybridization, we showed that *mcf2lb* is expressed in the pLLP during migration. Live imaging and subsequent 3D analysis of *mcf2lb* mutant pLLP cells showed disrupted apical constriction and subsequent rosette organization. This resulted in an excess number of deposited NMs along the trunk of the zebrafish. Cell polarity markers ZO-1 and Par-3 were apically localized, indicating that pLLP cells are properly polarized. In contrast, RhoA activity, as well as signaling components downstream of RhoA, Rock2a and non-muscle Myosin II, were diminished apically. Thus, Mcf2lb-dependent RhoA activation maintains the integrity of epithelial rosettes.

## Teaser

**Summary:** Mcf2lb, a RhoA GEF, regulates rosette integrity through modulating apical constriction of cells within rosettes during collective cell migration.

## INTRODUCTION

During development, cells undergo collective shape changes to accommodate organ morphogenesis. One prominent example of this behavior is in the formation of multicellular rosettes. Most multicellular rosettes are transient epithelial structures that contain five or more cells that interface at a central point, where apical membranes of these cells constrict. Multicellular rosettes are observed in many developmental contexts, including convergent extension during *Drosophila* embryogenesis, posterior Lateral Line (pLL) formation in zebrafish, vertebrate kidney tubule elongation, as well as numerous others (Blankenship et al., 2006; Gompel et al., 2001; Lienkamp et al., 2012).

Apical constriction is a process in which the apical portion of an epithelial columnar cell narrows while the base of the cell remains at a constant width. This process is dependent on the contraction of the acto-myosin network (Nishimura and Takeichi, 2008; Ernst et al., 2012; Harding and Nechiporuk, 2012). Before constriction, cells become polarized into apical and basal domains. Proper activation and distribution of the aPKC/Par-6/Par-3 polarity complex (McCaffrey and Macara, 2012) ensures the apical localization of cell junction proteins, including the tight junction protein ZO-1 (also known as TJP1A; Niessen, 2007). In addition, before constriction, the molecular players necessary for this process become apically localized, including F-actin fibers and non-muscle Myosin II.

To understand the molecular machinery that induces and maintains rosettes, we use a well-established model system, the posterior lateral line primordium (pLLP). The pLLP is a group of ∼150 cells that is organized into polarized rosettes; each rosette gives rise to a sensory organ on the surface of the trunk called a neuromast (NM). NMs are part of the lateral line mechanosensory system that detects changes in water current and controls various swimming behaviors (Coombs and Van Netten, 2005). During development, the pLLP forms immediately caudal to the otic vesicle. Between 22 and 48 hours post-fertilization (hpf), pLLP cells collectively migrate along the lateral aspect of the trunk (Dalle Nogare et al., 2017). Based on morphological and molecular differences, the migrating pLLP can be divided into two main regions, the leading region (leaders) and the trailing region (followers). Cells in the trailing region divide and differentiate during migration to form epithelial rosettes that are ultimately deposited (Lecaudey et al., 2008; Nechiporuk and Raible, 2008). This results in the deposition of five to six NMs along the trunk. Cells in the leading region remain undifferentiated throughout most of migration and eventually form two to three terminal NMs at the distal end of the trunk (Aman and Piotrowski, 2008). The proximity of the pLLP to the skin makes it highly amenable to high-resolution live imaging. Together with the genetic tractability of zebrafish, this makes the pLLP an attractive model for studying mechanisms of rosette formation *in vivo.*

Two complementary studies defined the molecular signaling pathways regulating rosette formation in both the leading and trailing cells of the pLLP (Ernst et al., 2012; Harding and Nechiporuk, 2012). Generally, Fgfr-Ras-MAPK signaling induces expression of Shroom3, a scaffold protein that binds Rho kinase (Rock2a), leading to its apical localization. RhoA activation of Rock2a induces phosphorylation of non-muscle Myosin II, initiating the constriction of apically localized actin fibers. However, how RhoA activity is regulated and maintained in this context is unknown.

In this study, we identified Mcf2lb as a regulator of rosette integrity during pLLP migration. We showed that loss of *mcf2lb* results in an excess number of NMs deposited along the trunk of the embryo. Using live imaging, we found that this phenotype results from deposition of rosette clusters in the mutant, instead of evenly spaced single rosettes in the wild type (WT). We further demonstrated that this behavior results from abnormal rosette organization in the *mcf2lb* mutant pLLP. 3D analysis of mutant cells revealed impaired apical constriction. Notably, the tight junction marker ZO-1 and polarity marker Par-3 remained apically localized, indicating that rosette cells are polarized. A fluorescent biosensor revealed an absence of RhoA signal at the rosette centers in *mcf2lb* mutants. Consistent with this observation, immunostaining of downstream RhoA signaling components, Rock2a and the non-muscle myosin II component Myosin Regulatory Light Chain (MRLC), showed diminished signal at the rosette centers in *mcf2lb* mutants. We propose a model whereby Mcf2lb acts as a guanine nucleotide exchange factor (GEF) to activate RhoA, which then initiates and/or maintains apical constriction.

## RESULTS

### Identification of factors that regulate actin dynamics in the pLLP

**Fig. 1.**
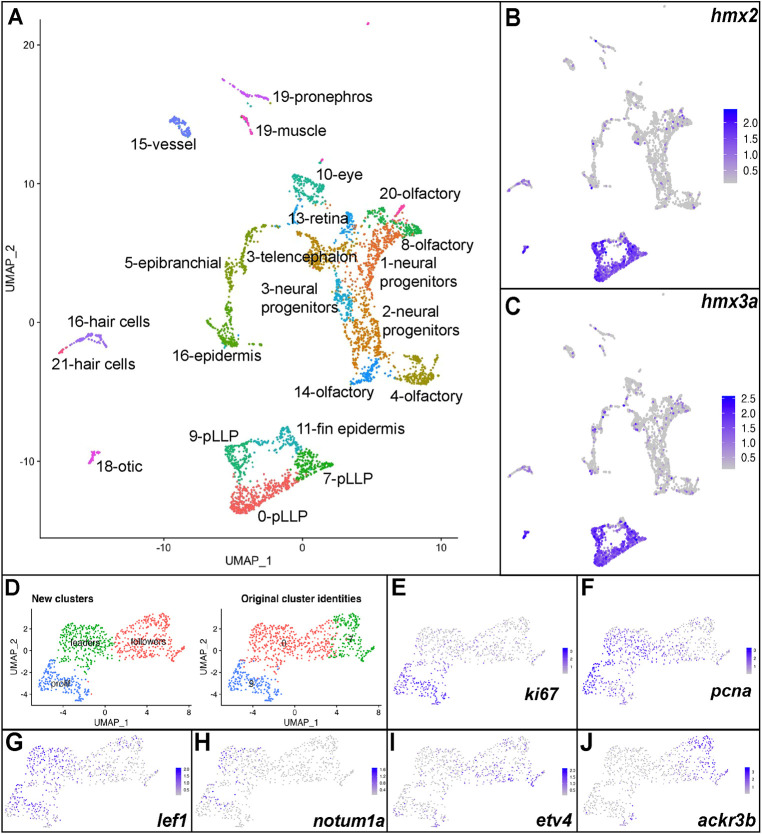
**Identification of the pLLP transcriptional profile by scRNA-seq.** (A) Unsupervised clustering and UMAP reduction diagram of cells derived from Tg(-8.0*claudinB: lynGFP)^zf106^*; TgBAC(*cxcr4b:F-tractin-mCherry)^p3^* embryos. (B,C) Feature plots of *hmx2* and *hmx3a* identify clusters 0, 7 and 9 as pLLP. (D) Unsupervised reclustering of clusters 0, 7 and 9. (E,F) Feature plots of *ki67* and *pcna* identify cluster 2 as proliferating cells. (G,H) Feature plots of *lef1* and *notum1a* identify cluster 1 as leader cells. (I,J) Feature plots of *etv4* and *ackr3b* identify cluster 0 as follower cells.

Owing to the important role of the actin cytoskeleton in both pLLP protrusive behavior as well as in the morphological changes that occur during rosette formation within the pLLP (Ernst et al., 2012; Harding and Nechiporuk, 2012; Dalle Nogare et al., 2020; Olson and Nechiporuk, 2021; Yamaguchi et al., 2022), we set out to identify potential genetic regulators of actin dynamics. To achieve this, we first identified the transcriptional profile of the pLLP by performing single-cell RNA-sequencing (scRNA-seq). We used fluorescence-activated cell sorting (FACS) to isolate cells from 30 hpf zebrafish embryos that carried two transgenes marking the pLLP, Tg(-8.0*claudinB: lynGFP)^zf106^* (Haas and Gilmour, 2006) and TgBAC(*cxcr4b:F-tractin-mCherry)^p3^* (Yamaguchi et al., 2022). GFP- and mCherry-positive cells were processed using the 10x Chromium platform, and libraries were sequenced at ∼25,000 reads per cell. scRNA-seq data were subjected to quality control and unsupervised clustering using the Seurat pipeline (Butler et al., 2018). Unsupervised clustering and UMAP reduction resulted in 22 individual clusters that are annotated in Fig. 1A. pLLP cells were identified as clusters expressing known lateral line markers, including *hmx2* and *hmx3a* (Fig. 1A-C) (Feng and Xu, 2010). Reclustering of pLLP cells did not reveal any additional transcriptional heterogeneity within the three pLLP clusters, although distribution of some cells in clusters 0 and 7 changed (Fig. 1D). Proliferation markers *ki67* (*mki67*) and *pcna* identified cluster 9 as proliferating pLLP cells (Fig. 1E,F) (Gerdes et al., 1984; Celis and Celis, 1985). Previous studies have shown that the Wnt signaling pathway is active in leading cells (Aman and Piotrowski, 2008). In contrast, downstream Fgf signaling components and the chemokine receptor *ackr3b* (also known as *cxcr7b*) are expressed in trailing pLLP cells (Raible and Brand, 2001; Dambly-Chaudière et al., 2007). *lef1* and *notum1a*, two Wnt pathway signaling components (Giráldez et al., 2002; Clevers, 2006), were upregulated in cluster 0, whereas *etv4* and *ackr3b* were upregulated in cluster 7 (Fig. 1G-J). Notably, proliferating cells expressed both leading and trailing cell markers (Fig. 1G,I). This is not surprising, as proliferation occurs throughout both the leading and the trailing regions of the pLLP (Dalle Nogare et al., 2017). Thus, our three subclusters mark transcriptionally distinct leader, follower and proliferating pLLP cells.

We next investigated expression of genes that regulate actin dynamics in pLLP cells. To achieve this, we used the Seurat function AddModuleScore to create a gene signature for Gene Ontology (GO) terms associated with actin regulation and actin dynamics (Ashburner et al., 2000). We then applied the clusterProfiler package to analyze these gene signatures in pLLP cells (Gene Ontology Consortium, 2021). This analysis showed that most of the GO term signatures were enriched in pLLP cells (Fig. S1A). Enrichment of components that regulate actin dynamics is also illustrated on the Regulation of Actin Cytoskeleton Kyoto Encyclopedia of Genes and Genomes (KEGG) pathway map (Fig. S1B) (Kanehisa et al., 2000). To visualize expression of individual genes, we grouped GO terms into: (1) actin binding; (2) Rho-GTPases, GAPs, GEFs; and (3) actin polymerization categories (Fig. S2). These analyses demonstrated that a vast majority of actin regulatory genes have higher levels of expression in the follower cell cluster in comparison with either the leader or the proliferating cell cluster (Figs S1A and S2). This observation is consistent with a recent study that assayed cytoskeleton-based stress forces along the migrating pLLP. It determined that the pLLP exerts higher stresses in the trailing rather than leading region (Yamaguchi et al., 2022).

### scRNA-seq validation by *in situ* hybridization

**Fig. 2.**
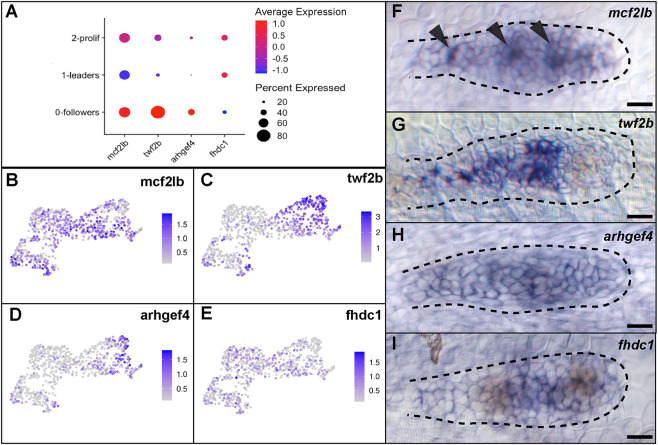
**Expression profile of genes that regulate actin dynamics.** (A) Dot plot expression profile of *mcf2lb*, *twf2b*, *arhgef4* and *fhdc1*. (B-E) Feature plots showing expression profile of the above four genes in pLLP clusters. (F-I) *In situ* hybridization of the four genes. Note that expression profiles via *in situ* hybridization largely match those observed by scRNA-seq. Arrowheads indicate rosette centers. Dotted lines indicate the outline of the pLLP. Scale bars: 10 μm.

To validate our scRNA-seq dataset, we used *in situ* hybridization to visualize expression of four genes, *mcf2lb*, *twf2b*, *arhgef4* and *fhdc1* in the pLLP. We chose these genes as they regulate different aspects of actin dynamics and show region-specific expression (Fig. 2). *mcf2lb* encodes a known GEF for RhoA, Rac1 and Cdc42 in different contexts, with the majority of the evidence supporting its role as a GEF for RhoA (Horii et al., 1994; Whitehead et al., 1999; Cheng et al., 2004). *In situ* hybridization for *mcf2lb* was consistent with the scRNA-seq: it is expressed throughout the pLLP, with lower levels in the leading region (Fig. 2A,B,F). Interestingly, the *mcf2lb* transcript appears to be enriched in the cells that form pLLP rosettes (Fig. 2F, arrowheads). *twf2b* encodes an F-actin capping protein that sequesters G-actin, thus inhibiting actin polymerization; it is expressed almost exclusively in the follower cells, as seen by both scRNA-seq and *in situ* (Fig. 2A,C,G) (Vartiainen et al., 2003; Nevalainen et al., 2009). *arhgef4* encodes a GEF for RhoA, Rac1 and Cdc42 and is upregulated in the followers of the pLLP via scRNA-seq. *In situ* hybridization revealed that *arhgef4* is mostly expressed throughout the pLLP, with some higher levels in the follower cells (Fig. 2A,D,H) (Kawasaki et al., 2000; Gotthardt and Ahmadian, 2007). Finally, *fhdc1* encodes an actin-binding protein involved in stress fiber formation; it is expressed higher in the leading cells, as seen by *in situ*, which is consistent with the scRNA-seq (Fig. 2A,E,I) (Young et al., 2008). In summary, *in vivo* expression patterns largely confirm those observed by scRNA-seq.

### Loss of *mcf2lb* results in the supernumerary deposition of NMs

Due to its role as a RhoA GEF and its rosette center-localized expression pattern, we hypothesized that *mcf2lb* may play a role in regulating rosette formation in the pLLP follower cells. To assess the role of *mcf2lb*, we generated two distinct *mcf2lb* mutant lines using CRISPR-Cas9-mediated gene editing (Fig. S3A). Both lines are presumably loss-of-function and do not exhibit any significant phenotypic differences from each other (Fig. S3B). Both zygotic and maternal zygotic homozygous *mcf2lb* mutants are viable and fertile. As *mcf2lb* is maternally contributed (Fig. S3C), it is not surprising that maternal zygotic *mcf2lb* mutants displayed a more severe phenotype than zygotic mutants (Fig. S3B and data below). Thus, we performed all our experiments in maternally zygotic homozygous mutants.

**Fig. 3.**
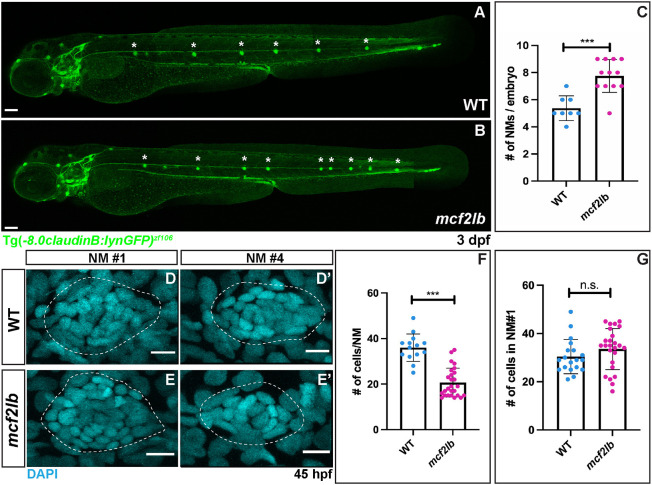
**Loss of Mcf2lb leads to the deposition of supernumerary NMs.** (A,B) Tg(-8.0*claudinB: lynGFP)*^zf106^ marks NMs in WT and *mcf2lb* mutant embryos at 3 dpf. Note the excess number of deposited trunk NMs in the *mcf2lb* mutants. Asterisks mark deposited trunk NMs. Full-body images in A were stitched together from four fields of view of individual body segments (head, trunk, tail) using the auto-blend feature in Photoshop. Full-body images in B were stitched together from three fields of view of the head and trunk using the auto-blend feature in Photoshop, and the tail image was manually included. (C) Mean number of NMs in WT (*n*=8 embryos) and *mcf2lb* mutant (*n*=12 embryos) embryos at 3 dpf. (D-E′) DAPI staining of nuclei in deposited NMs in WT (D,D′) and *mcf2lb* mutants (E,E′). (F) Mean number of cells per NM in WT (*n*=14 NMs from four embryos) and *mcf2lb* mutants (*n*=26 NMs from five embryos). (G) Mean number of cells in the first NM in WT (*n*=19 NMs) and *mcf2lb* mutants (*n*=25 NMs). ***P<0.001 (unpaired two-tailed *t*-test). n.s., not significant. Error bars are s.d. Scale bars: 100 μm (A,B); 5 μm (D,E).

We next used the Tg(-8.0*claudinB: lynGFP)^zf106^* transgene to assess the gross morphology of the pLL in the *mcf2lb* mutants. At 3 days post-fertilization (dpf), *mcf2lb* mutant embryos showed an excess number of deposited trunk NMs compared with control embryos (Fig. 3A-C; mean number of NMs in WT=5.375 versus *mcf2lb* mutants=7.750; *P*<0.001, unpaired two-tailed *t*-test). Owing to the increase in the number of deposited NMs, we asked whether the size of the NMs was different between WT and *mcf2lb* mutants. Based on nuclei staining, *mcf2lb* mutants had significantly fewer NM cells than WT (Fig. 3D-F; mean size of NMs in WT=36 cells versus *mcf2lb* mutant=20.65 cells; *P*<0.001, unpaired two-tailed *t*-test). Interestingly, no significant difference was observed in the size of the first deposited NM when comparing WT with *mcf2lb* mutants (Fig. 3D,E,G; WT=30.42 cells versus *mcf2lb* mutant=33.86 cells; *P*=0.20, unpaired two-tailed *t*-test). As the rostral-most pLLP rosette (first proto-NM) is patterned before the onset of migration (Nechiporuk and Raible, 2008), this indicates that *mcf2lb* does not play a role in regulating NM size until pLLP migration begins.

### *mcf2lb* mutants show impaired pLLP deposition behavior and abnormal pLLP organization

**Fig. 4.**
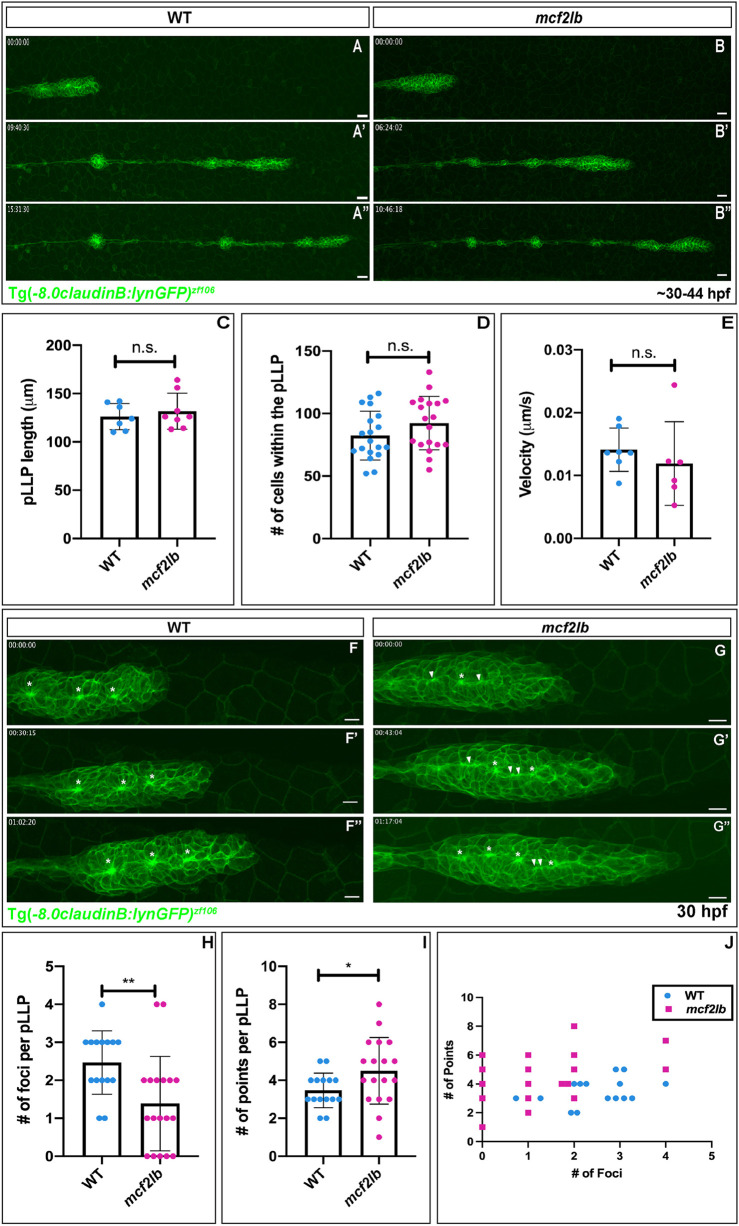
***mcf2lb* mutants show abnormal organization of the pLLP and NM deposition.** (A-B″) Lateral views of stills from time-lapse movies obtained between 30 and 44 hpf of either WT or *mcf2lb* mutants positive for Tg(-8.0*claudinB: lynGFP)^zf106^* (Movies 1,2). (C) Mean pLLP length in WT (*n*=7 pLLPs) and *mcf2lb* mutants (*n*=8 pLLPs). (D) Mean number of cells within the pLLP in WT (*n*=19 pLLPs) and *mcf2lb* mutants (*n*=19 pLLPs). (E) Mean velocity of the pLLP in WT (*n*=7 pLLPs) and *mcf2lb* mutants (*n*=6 pLLPs). (F-G″) High-magnification lateral views of still images from movies of the migrating pLLP in WT and *mcf2lb* mutants (Movies 3,4). pLLPs were imaged for ∼1.5 h starting at ∼30 hpf. Note the abnormal organization of the pLLP in *mcf2lb* mutants and the beads on a string appearance along the midline instead of distinct clusters of membrane as in WT (Movies 3,4). Asterisks indicate foci. Arrowheads indicate points. (H) Mean number of foci per pLLP in WT (*n*=15 pLLPs) and *mcf2lb* mutants (*n*=18 pLLPs). (I) Mean number of points per pLLP in WT (*n*=15 pLLPs) and *mcf2lb* mutants (*n*=18 pLLPs). (J) Scatter plot of foci numbers per pLLP versus number of points per pLLP in WT (*n*=15 pLLPs) and *mcf2lb* mutants (*n*=18 pLLPs). *P<0.05, **P<0.01 (unpaired two-tailed *t*-test). n.s., not significant. Error bars are s.d. Scale bars: 20 μm (A,B); 10 μm (F,G).

To address the cellular mechanism leading to extra NMs, we assayed NM deposition and pLLP organization in *mcf2lb* mutants. To achieve this, we imaged pLLP migration in WT or *mcf2lb* mutant Tg(-8.0*claudinB: lynGFP)^zf106^*-positive embryos between 30 and 44 hpf (Fig. 4A,B; Movie 1,2). *mcf2lb* pLLP deposited multiple rosettes concurrently, instead of individual rosettes five to seven somites apart as in WT embryos. These groups of rosettes were often deposited as one large cluster of cells that then resolved into individual rosettes over time (Fig. 4B; Movie 2). Despite this abnormal behavior, velocity of the mutant pLLP did not differ from that of the WT (Fig. 4E; mean velocity WT=0.014 μm/s versus *mcf2lb* mutant=0.012 μm/s; *P*=0.45, unpaired two-tailed *t*-test). In addition, there was no difference in the pLLP length (Fig. 4C; mean length WT=126.1 μm versus *mcf2lb* mutant=131.8 μm; *P*=0.52, unpaired two-tailed *t*-test) or the number of cells within the migrating pLLP (Fig. 4D; mean number of cells WT=82.47 cells versus *mcf2lb* mutant=92.37 cells; *P*=0.14, unpaired two-tailed *t*-test) when comparing WT with *mcf2lb* mutants. These results indicate that, although the pLLP size and migration are not perturbed in *mcf2lb* mutants, rosette deposition behavior is impaired.

Abnormal rosette deposition behavior implied that pLLP organization might be abnormal in *mcf2lb* mutants. To examine pLLP organization, we imaged pLLPs in either WT or *mcf2lb* mutant Tg(-8.0*claudinB: lynGFP)^zf106^*-positive embryos at high magnification during migration. This revealed abnormal organization of rosettes in the pLLP of *mcf2lb* mutants (Fig. 4F,G; Movie 3, 4). WT pLLP usually contained two or three rosettes that display prominent gatherings of apical membrane, which we termed foci (Fig. 4F; Movie 3). In contrast, *mcf2lb* mutants displayed a ‘beads on a string’ appearance of constricted membranes: the foci are not distinct and instead vary in size and shape (Fig. 4G; Movie 4). To quantify this pLLP phenotype, we divided the gatherings of membrane into two categories, either foci or points. Gatherings of membrane were deemed to be foci if the width and the height of the focus were within one standard deviation above or below the mean width and height of all foci measured in WT. If it did not meet those criteria, it was deemed a point. In panels Fig. 4F and G, asterisks depict foci and arrowheads depict points. On average, *mcf2lb* mutants contained significantly fewer foci (Fig. 4H; WT=2.47 foci versus *mcf2lb* mutant=1.39 foci; *P*=0.005, unpaired two-tailed *t*-test) and a significantly greater number of points (Fig. 4I; WT=3.47 versus *mcf2lb* mutant=4.50; *P*=0.049, unpaired two-tailed *t*-test) than WT pLLP. Additionally, as the number of points increased in *mcf2lb* mutant pLLP the number of foci decreased (Fig. 4J). These results indicate that *mcf2lb* mutant pLLPs have abnormal cellular organization and a diminished ability to form proper rosettes.

### Rosette and hair cell morphology is altered in *mcf2lb* mutants

Rosette formation is dependent on the activation of Fgf signaling in the trailing cells (Nechiporuk and Raible, 2008; Harding and Nechiporuk, 2012). Subsequent rosette morphogenesis is linked to the expression of *atoh1a* in hair cell progenitors within each rosette (Lecaudey et al., 2008; Nechiporuk and Raible, 2008). As we observed abnormal rosette formation in *mcf2lb* mutants, we asked whether activation of Fgf signaling and formation of *atoh1a*-positive foci are also affected. We observed no difference in the expression of *etv4*, a direct transcriptional target of Fgf signaling (McCabe et al., 2006), between *mcf2lb* mutants and wild-type controls (Fig. S4A,B,G; mean ratio of *etv4* domain to pLLP length in WT=0.601 versus *mcf2lb* mutant=0.584; *P*=0.649, unpaired two-tailed *t*-test). However, we did find a significant increase in the number of *atoh1a-*positive foci in mutant pLLPs (Fig. S4C,D,H; mean number of foci in WT=2.727 versus *mcf2lb* mutant=3.636; *P*=0.0219, unpaired two-tailed *t*-test). *deltaA* is a Notch ligand that is also expressed in hair cell precursors and acts downstream of *atoh1a*. We found no significant differences in the number of *deltaA-*positive foci (Fig. S4E,F,I; mean number of foci in WT=2.800 versus *mcf2lb* mutant=2.952; *P*=0.814, Mann–Whitney *U-*test). Interestingly, there was an increase in the number of *deltaA*-positive cells per rosette center in *mcf2lb* mutants in comparison with WT, although this result was not statistically significant (Fig. S4F,J; mean number of cells per foci in WT=1.567 cells versus *mcf2lb* mutant=2.151 cells; *P*=0.1042, unpaired two-tailed *t*-test). Altogether, these results indicate that, although the Fgf signaling domain is not changed, the number of *atoh1a*-positive foci that organize rosette morphogenesis is increased.

Following migration, *atoh1a* and *deltaA* continue to express in hair cell precursors within the deposited NMs in both WT and *mcf2lb* mutants (Fig. S5A-D). However, the hair cell morphology was altered in *mcf2lb* mutants (Fig. S5E-I). Specifically, there was a significant decrease in the basal width of the hair cells (Fig. S5I; WT=7.26 μm versus *mcf2lb* mutant=5.31 μm; *P*=0.001, unpaired two-tailed *t*-test); although both hair cell volume and apical width were not altered in mutant hair cells (Fig. S5G,H). These results indicate that despite NM hair cells differentiating properly, their morphology is altered.

### *mcf2lb* mutants show impaired apical constriction and rosette integrity in the pLLP

**Fig. 5.**
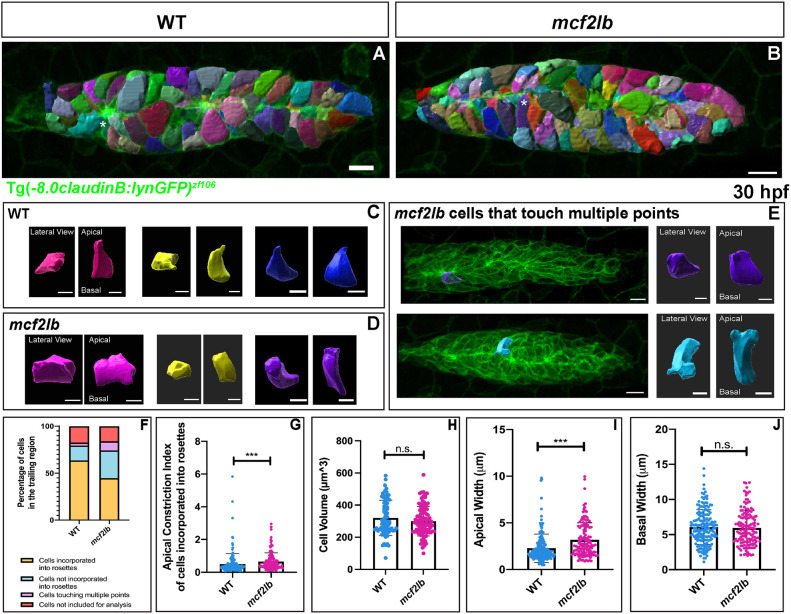
***mcf2lb* mutants show impaired apical constriction of cells incorporated into rosettes.** (A,B) Cellular reconstruction of WT and *mcf2lb* mutant pLLPs. (C,D) Examples of apically constricted cells from WT (C) and *mc2lb* mutant (D) pLLPs. The left side panels show the lateral (top-down) view as in panels A and B, and the right panels show the apical/basal views of the cell (cell is virtually turned by 90°). Blue and purple cells marked by the asterisks in panels A and B are also shown in C and D, respectively. (E) Examples of cells that are making contact with multiple points in *mcf2lb* mutants. Left panel is a lateral (top-down) view; right panel is an apical/basal view. (F) Categorical breakdown of cells in the trailing region of WT (*n*=258 cells from four pLLPs) and *mcf2lb* mutants (*n*=311 cells from four pLLPs). Note that in *mcf2lb* mutants, there is an increase in the percentage of cells that touch multiple points and an increase in the percentage of cells not incorporated into rosettes. Subsequently, there is a decrease in the percentage of cells incorporated into rosettes. (G) Mean apical constriction index of cells that are incorporated into rosettes in WT (*n*=169 cells from four pLLPs) and *mcf2lb* mutants (*n*=139 cells from four pLLPs). (H) Mean cell volume of cells incorporated into the trailing rosette in WT (*n*=74 cells from four pLLPs) and *mcf2lb* mutants (*n*=91 cells from four pLLPs). (I) Mean apical width of cells incorporated into rosettes in WT (*n*=169 cells from four pLLPs) and *mcf2lb* mutants (*n*=139 cells from four pLLPs). (J) Mean basal width of cells incorporated into rosettes in WT (*n*=169 cells from four pLLPs) and *mcf2lb* mutants (*n*=139 cells from four pLLPs). Note that the apical width is significantly increased in *mcf2lb* mutants whereas there is no significant difference in the basal width. ***P<0.001 (F: chi-square test; G,I,J: Mann–Whitney *U*-test; H: unpaired two-tailed *t*-test). n.s., not significant. Error bars are s.d. Scale bars: 10 μm (A,B and E pLLP); 5 μm (C,D and E individual cells).

Given the observed rosette disorganization and enhanced expression of *mcf2lb* in rosette centers, we hypothesized that apical constriction could be impaired in *mcf2lb* mutants. To examine this, we used Imaris to reconstruct surfaces of all cells in the pLLP in both WT and *mcf2lb* mutants (Fig. 5A,B). Following 3D cellular reconstruction, we examined the morphology of cells within the trailing region of the pLLP and found that many of these cells were not apically constricted in the *mcf2lb* mutants (Fig. 5C,D). In addition, although in WT pLLP cells usually constrict to an individual focus, we found that in *mcf2lb* mutants some cells made contact with multiple points (Fig. 5E). To characterize these deficiencies, we divided cells into four categories: incorporated into rosettes, not incorporated into rosettes, touching multiple points and those we could not include in analysis (dividing cells and cells that were not columnar). In *mcf2lb* mutants, the proportion of cells that was incorporated into rosettes was diminished in comparison with WT pLLP, whereas the percentage of cells that was not incorporated into rosettes was expanded (Fig. 5F; incorporated into rosettes: WT pLLP=64% versus *mcf2lb* mutant pLLP=45%; not incorporated into rosettes: WT pLLP=16% versus *mcf2lb* mutant pLLP=30%; *P*<0.001, chi-square test). Additionally, the percentage of cells touching multiple points was increased in *mcf2lb* mutant pLLP in comparison with WT pLLP (Fig. 5F; WT pLLP=3% versus *mcf2lb* mutant=10%; *P*<0.001, chi-square test). To quantify apical constriction, we measured the apical constriction index (ACI) of cells incorporated into rosettes in the trailing region of both WT and *mcf2lb* mutant pLLPs. In *mcf2lb* mutants, the mean ACI of cells incorporated into rosettes was significantly increased in comparison with WT pLLP (Fig. 5G; WT=0.50 versus *mcf2lb*=0.65; *P*<0.001, Mann–Whitney *U-*test). To further examine this impairment, we also compared the mean apical width and basal width of cells incorporated into rosettes. We found a significant increase in the apical width of *mcf2lb* mutant cells (Fig. 5I; WT=2.28 μm versus *mc2lb* mutants=3.18 μm; *P*<0.001, Mann–Whitney *U-*test). However, there was no significant difference in the basal width of cells when comparing WT with *mcf2lb* mutants (Fig. 5J; WT=6.06 μm versus *mc2lb* mutants=5.94 μm; WT; *P*=0.55 Mann–Whitney *U-*test). Additionally, the volume of cells incorporated into the trailing-most rosette did not significantly differ between WT and *mcf2lb* mutant pLLP (Fig. 5H; WT=320.0 µm^3^ versus *mcf2lb* mutants=300.1 µm^3^; *P*=0.208, unpaired two-tailed *t*-test). These results indicate that the ability of cells incorporated into rosettes to apically constrict is impaired in *mcf2lb* mutants; however, the overall size and basal width of cells are unchanged.

Previous studies have revealed that the rostral-most rosette in the pLLP forms a microlumen within its apical region (Durdu et al., 2014). Secreted Fgf ligands accumulate in the microlumen and maintain rosette integrity and orderly NM deposition (Durdu et al., 2014). After rosette deposition, the microlumen eventually expands into a lumen housing hair cell stereo- and kinocilia. To assess whether microlumen formation is affected in *mcf2lb* mutants, we transiently expressed secreted GFP (secGFP) in the migrating pLLP as well as newly deposited NMs. In WT controls, even a single cell with secGFP expression was sufficient to mark the microlumen in the trailing rosettes and recently deposited NMs (Fig. S6A,C). In contrast, we detected low secGFP in *mcf2lb* mutants (Fig. S6A-E; mean normalized fluorescence in WT=0.08433 versus *mcf2lb* mutant=0.01883; *P*<0.001, unpaired two-tailed *t*-test). These data indicate that integrity is compromised in the *mcf2lb* mutant in the trailing rosette and, consequently, NM.

### *mcf2lb* mutants display abnormal apical membrane dynamics

**Fig. 6.**
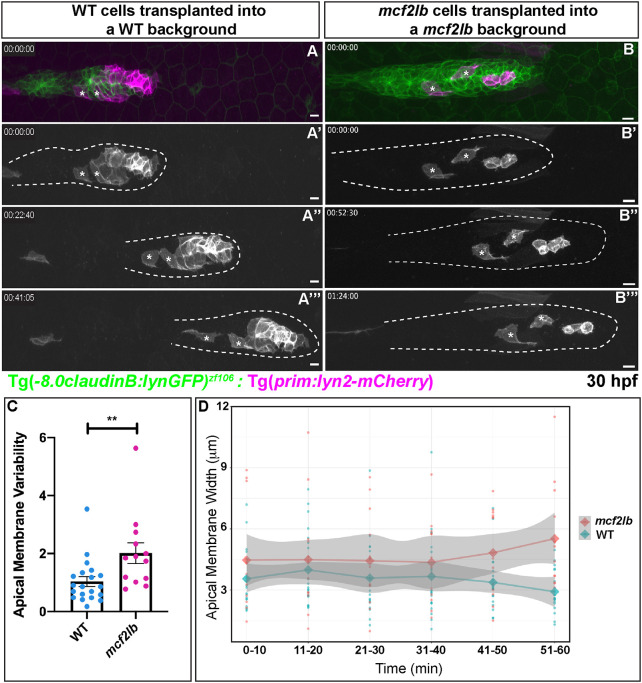
***mcf2lb* mutants show greater variability in apical membrane dynamics.** (A-B‴) Donor cells derived from either WT or *mcf2lb* mutant Tg(*prim:lyn2-mCherry*) (magenta) were transplanted into WT or *mcf2lb* mutant Tg(-8.0*claudinB: lynGFP*)^zf106^-positive (green) embryos, respectively, and mounted for live imaging between 30 and 32 hpf (Movie 5,6). Tg(*prim:lyn2-mCherry*) cells are shown in grayscale for clarity. Asterisks indicate cells used for analysis. (C) Mean membrane variability of transplanted WT (*n*=22 cells from seven embryos) or *mcf2lb* mutant (*n*=13 cells from three embryos) cells over time from movies obtained from experiments in panels A and B. Membrane variability is defined as the standard deviation of the apical width of a cell over an hour period of imaging in apically constricted cells. Error bar is s.d. (D) Apical membrane width over time with s.e.m. (shaded area). Note higher variability in the mutant. **P<0.01 (Mann–Whitney *U-*test). Scale bars: 10 μm.

In order to visualize the dynamics of apical membranes in single pLLP cells, we generated mosaic primordia that contained a few cells with fluorescently labeled membranes. To achieve this, we transplanted a small number of cells from a donor embryo expressing the Tg(*prim:lyn2-mCherry*) transgene into a Tg(-8.0*claudinB: lynGFP)^zf106^* host. Observation of WT cells in a WT background revealed that, once incorporated into a rosette, apically constricted cells maintained contact with the rosette center during migration (Fig. 6A; Movie 5). Interestingly, we observed both apical membrane expansion and contraction in apically constricted cells both in WT and mutant pLLPs. To quantify apical membrane dynamics, we measured ‘average membrane variability’ and average apical membrane width (Fig. 6C,D). We defined ‘average membrane variability’ as the standard deviation of the apical width of a cell over the course of 1 h imaging period. We found that apical membranes of *mcf2lb* mutant cells transplanted into the mutant background are much more variable than those of WT cells over the course of the imaging period (Fig. 6B-D; Movie 6). When quantified, the ‘average membrane variability’ in WT cells was 1.19 μm versus 2.01 μm in *mcf2lb* mutant cells (*P*=0.01, Mann–Whitney *U-*test). These results indicate that *mcf2lb* mutant cells cannot maintain an apically constricted state.

### Cell polarity is normal in *mcf2lb* mutant pLLP

**Fig. 7.**
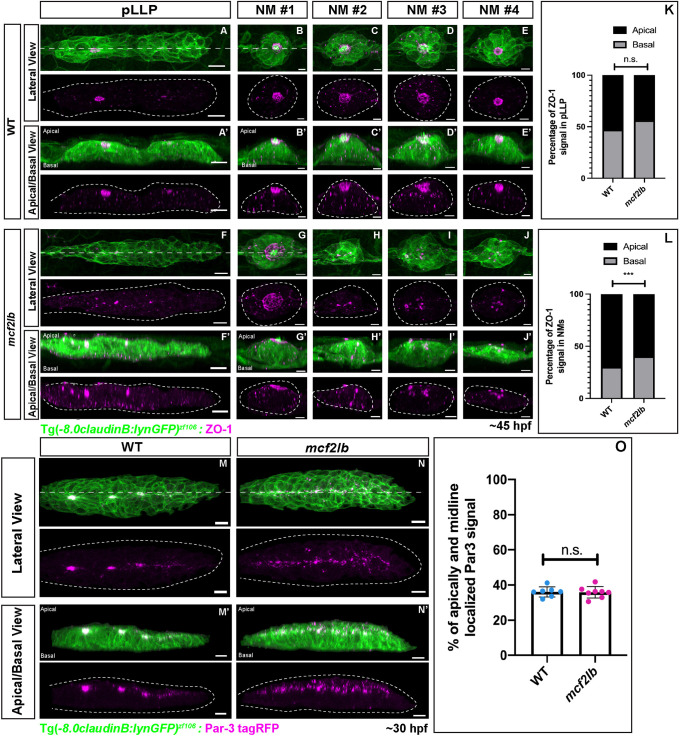
**pLLP cell polarity is largely unaffected in *mcf2lb* mutants.** (A-J′) Immunostaining for the tight junction marker ZO-1 in WT and mutant pLLPs at 45 hpf. Panels A-J show the lateral (top-down) view, A′-J′ show an apical-basal view (images were digitally rotated 90° around *x*-axis; dashed lines in A,B,F,G). All images are *z*-projections. Dotted lines indicate the pLLP in B and F and NMs in B-E and G-J. Note the ring structure and the apical localization of ZO-1 in the trailing-most rosette and deposited NMs in WT. In contrast, ZO-1 signal is disorganized in *mc2lb* mutants. (K) Quantification of the percentage of apical- and basal-localized ZO-1 signal in the pLLP in WT (*n*=8 pLLPs) and *mcf2lb* mutants (*n*=13 pLLPs). (L) Quantification of the percentage of apical- and basal-localized ZO-1 signal in NMs in WT (*n*=15 NMs from four embryos) and *mc2lb* mutants (*n*=26 NMs from five embryos). (M-N′) Par-3-tagRFP expression in WT and *mcf2lb* mutant pLLPs. Panels M and N show the lateral (top-down) view, panels M′ and N′ show an apical-basal view (images were digitally rotated 90° around *x*-axis; dashed lines M,N). All images are *z*-projections. Note Par-3 localization to the rosette centers and the midline in WT embryos; in contrast, Par-3 is localized to the midline but not organized into rosette centers in *mcf2lb* mutants. Par-3 is apically localized in both WT and *mcf2lb* mutant pLLPs. (O) Quantification of the percentage of apically- and midline-localized Par-3 signal in WT (*n*=7 pLLPs) and *mcf2lb* mutants (*n*=8 pLLPs). ***P<0.001 (unpaired two-tailed *t*-test). n.s., not significant. Error bars are s.d. Scale bars: 10 μm (A,F,M,N); 5 μm (B-D,G-J).

As *mcf2lb* mutant cells show impairment in apical constriction, we next asked whether mutant cells were properly polarized. To examine this, we visualized the localization of the tight junction scaffolding protein ZO-1 in WT and *mcf2lb* mutant pLLPs at 45 hpf (Niessen, 2007). In WT pLLP, ZO-1 immunostaining showed enhancement at the rosette centers and formation of a ring-like structure in the caudal-most rosette (Fig. 7A). After digitally rotating the pLLP 90° along the *x*-axis, ZO-1 immunostaining appeared to be apically localized at the rosette centers (Fig. 7A′). In *mcf2lb* mutant pLLP, ZO-1 remained localized to the midline; however, there was no ring-like structure present in the most caudal rosette. Instead, ZO-1 staining was more punctate (Fig. 7F). Nevertheless, ZO-1 remained apically localized in *mcf2lb* mutants (Fig. 7F′,K; percentage of ZO-1 signal apically localized: WT=53% versus *mcf2lb* mutant=44%; *P*=0.17, unpaired two-tailed *t*-test). These results indicate that, although the organization of ZO-1 is impaired, mutant pLLP cells are polarized.

In addition to ZO-1, we also examined the localization of the polarity marker Par-3. Par-3 is a component of the aPKC complex that assembles apically to tight junctions in epithelial cells (McCaffrey and Macara, 2012). To visualize Par-3 localization, we injected *par3-tagRFP* RNA into either WT or *mcf2lb* mutant Tg(-8.0*claudinB: lynGFP)^zf106^*-positive embryos, and the injected embryos were mounted for live imaging at 30 hpf. Par-3 localized to the rosette centers in the migrating WT pLLP. Digital rotation of the images by 90° along the *x*-axis revealed Par-3 apical localization (Fig. 7M,M′). In *mcf2lb* mutant pLLP, Par-3 localized entirely along the pLLP midline instead of rosette centers. Digital rotation of the images by 90° along the *x*-axis revealed proper apical localization of Par-3 (Fig. 7N-O; percentage of Par-3 signal that is midline and apically localized: WT=36% versus *mcf2lb* mutant=36%; *P*=0.88, unpaired two-tailed *t*-test). These results confirmed that mutant pLLP cells are apically polarized.

Finally, we examined ZO-1 immunostaining in deposited NMs at 45 hpf in WT and *mcf2lb* mutants. As NMs mature, a ring-like opening forms at the center of a NM to allow for stereociliary bundles to push through (Fig. 7B′). In WT embryos, all deposited NMs had a ring of ZO-1 that was apically localized (Fig. 7B-E). In *mcf2lb* mutants, this ring structure was only observed in NM1 (Fig. 7G). The remaining NMs showed disorganized ZO-1 staining and impaired apical localization of ZO-1 (Fig. 7H-J,L; percentage of signal apically localized WT=69% versus *mcf2lb* mutant=60%; *P*<0.001, unpaired two-tailed *t*-test). These results indicate that abnormal apical constriction in mutant rosettes ultimately leads to the formation of disorganized NMs.

### RhoA signaling is disrupted in *mcf2lb* mutant pLLP

**Fig. 8.**
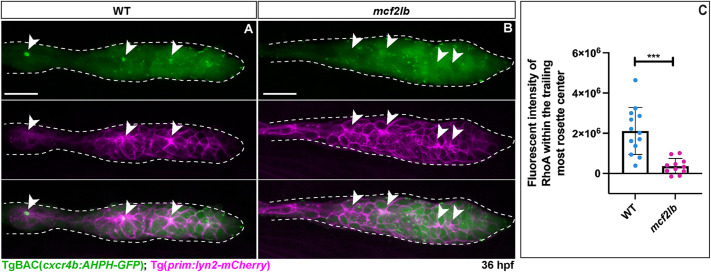
**RhoA signal is lost at rosette centers in *mcf2lb* mutants.** (A,B) Fluorescent signal from the RhoA sensor line TgBAC(*cxcr4b:AHPH-GFP*) at 36 hpf in WT (A) and the *mcf2lb* mutant (B). Tg(*prim:lyn2-mCherry)* marks cell membranes in the migrating pLLP. Dashed lines mark the pLLP. Arrowheads indicate rosette centers and points. Note the absence of RhoA signal at the rosette centers and points in the *mcf2lb* mutant pLLP. (C) Quantification of RhoA signal at the center of the trailing-most rosette (point) within the migrating pLLP in WT (*n*=13) and *mcf2lb* mutants (*n*=10). ***P<0.001 (unpaired two-tailed *t*-test). Error bars are s.d. Scale bars: 20 μm.

Mcf2l acts as a RhoA GEF in cultured mammalian cells and cortical neurons (Whitehead et al., 1999; Hayashi et al., 2013). As the RhoA pathway is required for apical constriction of pLLP cells (Harding and Nechiporuk, 2012), we examined RhoA signaling in the mutant pLLP using the transgenic RhoA biosensor line TgBAC(*cxcr4b:AHPH-GFP*) (Qian et al., 2023 preprint). In this transgene, the RhoA binding domain of the RhoA effector Anillin is fused to GFP; thus, higher fluorescence of this sensor corresponds to recruitment and/or stabilization of active RhoA (Piekny and Glotzer, 2008). Live imaging revealed an enhanced RhoA signal at the rosette centers within the WT pLLP as well as in the depositing NM (Fig. 8A). In contrast, we observed more than a 5-fold reduction in RhoA signal at the presumptive rosette centers of *mcf2lb* mutants (Fig. 8B,C; total fluorescence intensity: WT=2,113,951 versus *mcf2lb=*361,823; *P*<0.001, unpaired two-tailed *t*-test). These results indicate that there is a significant reduction in the amount of active RhoA at the rosette centers in *mcf2lb* mutants.

**Fig. 9.**
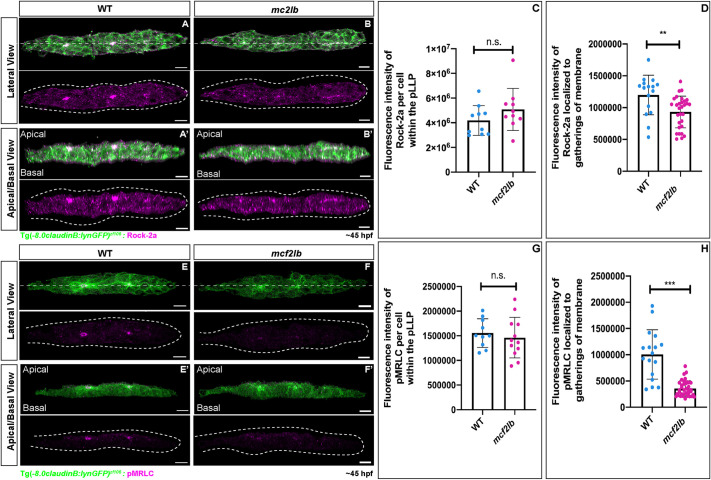
**RhoA signaling is disrupted in *mcf2lb* mutants.** (A-B′) Immunostaining for Rock2a in WT and *mcf2lb* mutants. A,B show the lateral view (top-down); A′,B′ show the apical-basal view (images were virtually rotated 90° around the *x*-axis; dashed lines A,B). All images are *z*-projections. Images are masked to show signal within the pLLP. Note the localization of Rock2a is diminished in *mcf2lb* mutant pLLP. (C) Total fluorescence of Rock2a per cell in WT (*n*=10 pLLPs) and *mcf2lb* mutants (*n*=10 pLLPs). (D) Fluorescence of Rock2a at rosette centers in WT (*n*=16 points from ten pLLP) and *mcf2lb* mutants (*n*=30 points from ten pLLPs). (E-F′) Immunostaining for pMRLC in WT and *mcf2lb* mutants. E,F show the lateral (top-down) view; E′,F′ show the apical-basal view (images were virtually rotated 90° around the *x*-axis; dashed lines E,F). All images are *z*-projections. Images are masked to show signal within the pLLP. Note diminished localization of pMRLC in the *mcf2lb* mutant pLLPs. (G) Total fluorescence of pMRLC per cell in WT (*n*=10 pLLPs) and *mcf2lb* mutants (*n*=13 pLLPs). (H) Fluorescence of pMRLC at the gatherings of the membranes in WT (*n*=17 points from ten pLLPs) and *mcf2lb* mutants (*n*=37 points from 13 pLLPs). Dotted lines indicate pLLP. **P<0.01, ***P<0.001 (unpaired two-tailed *t*-test). n.s., not significant. Error bars are s.d. Scale bars: 10 μm.

Next, we examined the signaling components downstream of RhoA necessary for apical constriction. In the pLLP, RhoA activates the Rho kinase Rock2a, which is apically scaffolded by Shroom3 (Ernst et al., 2012; Harding and Nechiporuk, 2012). Rock2a then phosphorylates MRLC, which activates non-muscle myosin II-mediated apical constriction (Harding and Nechiporuk, 2012). We first examined whether Rock2a was present and properly localized at rosette centers. Overall levels of Rock2a were not significantly different between WT and *mcf2lb* mutant pLLPs (Fig. 9A-D; mean fluorescence of Rock2a per cell: WT pLLP=4,185,000 versus *mcf2lb* mutant pLLP=5,080,000; *P*=0.19, unpaired two-tailed *t*-test). However, there was a significant difference in fluorescence intensity at the rosette centers when comparing WT with *mcf2lb* mutants (Fig. 9A-D; mean fluorescence of Rock2a at rosette centers: WT=1,198,500 versus *mcf2lb* mutant=931,700; *P*=0.003, unpaired two-tailed *t*-test). To examine apical localization of Rock2a, images were digitally rotated 90° along the *x*-axis. In WT and *mcf2lb* mutant pLLPs, Rock2a was localized apically (Fig. 9A′,B′). These results demonstrate that, although Rock2a levels and its apical localization are not changed, there is a decreased amount of Rock2a at the rosette centers in *mcf2lb* mutants.

We then asked whether a downstream effector of Rock2a, the MRLC component of non-muscle myosin II, is activated in mutant pLLP. In WT pLLP, phosphorylated MRLC (pMRLC) accumulated at rosette centers (Fig. 9E). However, in *mcf2lb* mutants, pMRLC signal was diminished at rosette centers (Fig. 9F,H; WT=1,005,529 versus *mcf2lb*=357,595; *P*<0.001, unpaired two-tailed *t*-test). To examine apical localization of pMRLC, images were digitally rotated 90° along the *x*-axis. In both WT and *mcf2lb* mutant pLLPs, pMRLC was apically localized (Fig. 9E′,F′). In summary, these data show that RhoA activation is strongly reduced in the *mcf2lb* mutants, which in turn prevents activation of RhoA downstream signaling responsible for apical constriction.

## DISCUSSION

Using scRNA-seq, we defined a comprehensive set of genes that regulate the actin cytoskeleton during pLLP migration. We then focused on *mcf2lb* and showed that it is required for apical constriction and rosette integrity during pLLP migration. We propose that Mc2lb activates RhoA, which subsequently activates Rock2a; Rock2a then phosphorylates non-muscle myosin II and induces apical constriction through the interaction of non-muscle myosin II with apically localized actin fibers. Notably, we still observed a partial constriction of pLLP cells despite an almost complete absence of RhoA activation. This observation argues that multiple factors act in parallel to mediate apical constriction in the pLLP. One possible candidate is the small GTPase Cdc42, which can activate a myosin light-chain kinase dependent apical constriction independent of RhoA (Marston et al., 2016; Zihni et al., 2017).

Is it possible that in addition to its role in regulating RhoA during apical constriction, Mcf2lb may be mediating other cellular processes necessary for pLLP differentiation and/or migration? We found that migration speed was unchanged in *mcf2lb* mutants, implying that protrusive behavior is normal. We also showed that pLLP cells are polarized and we did not observe any phenotypes previously associated with the loss of cellular adhesion (Colak-Champollion et al., 2019; Matsuda and Chitnis, 2010). Finally, mutant pLLPs have the same cell number when compared with WT pLLPs, arguing that proliferation and survival is normal as well. Altogether, this indicates that in the pLLP, Mcf2lb is specifically involved in maintaining apical constriction rather than regulating other cellular processes.

One future question is what regulates the expression of *mcf2lb* in the pLLP? Previous studies showed that Fgf/Notch signaling is required for localization of Rock2a and pMRLC via Shroom3 to the apical region of pLLP cells (Ernst et al., 2012; Harding and Nechiporuk, 2012; Kozlovskaja-Gumbriene et al., 2017). However, we found that *mcf2lb* expression is independent of Fgf signaling (data not shown). Thus, a pathway other than Fgf must maintain *mcf2lb* expression.

### Improper apical constriction results in impaired rosette integrity and rosette deposition

Regular deposition of rosettes from the pLLP is necessary for the even spacing of pLL mechanosensory organs along the trunk. In *mcf2lb* mutants, we observe abnormal spacing of primary NMs, which results from the deposition of large groups of unclustered cells that appear to lack cohesion; although, these cells ultimately resolve into two or three NMs. These results suggest that adhesion may be dysregulated during the initial deposition, which is ultimately resolved. Despite this deposition phenotype, the mutant pLLP maintains its normal size and migrates collectively, suggesting that the deposition mechanisms per se are not disrupted, but rather the ability of the mutant pLLP to deposit stable rosettes at regular intervals. The latter mechanism is cell autonomous to the pLLP and not induced by external factors (Aman et al., 2011; Durdu et al., 2014). Active Fgf signaling in the trailing cells is required for this process (Durdu et al., 2014). Reducing Fgf signaling through the use of the Fgf inhibitor SU5402 resulted in a dose-dependent delay in NM deposition, whereas increasing Fgf activity through overexpression of the Fgf ligand Fgf3 resulted in an increased rate of NM deposition (Durdu et al., 2014). However, in contrast to the *mcf2lb* mutant phenotype, this higher Fgf level did not result in more trunk NMs compared with controls. This led to the hypothesis that Fgf activity within the trailing pLLP can control the frequency of NM deposition. As Fgf ligands are concentrated in the microlumen of the trailing-most rosette (Durdu et al., 2014), disruption of this microlumen will affect the regular deposition of NMs (Durdu et al., 2014). Our experiments with secGFP implied that the microlumen is indeed disrupted in *mcf2lb* mutants; however, additional experiments visualizing microlumen integrity via transmission electron microscopy would be necessary to unambiguously confirm this observation. As Fgf ligands are concentrated in the microlumen and signal to adjacent cells, its disruption may lead to more cells coming into contact with Fgf ligands. This disruption in Fgf signaling domains could explain the altered expression patterns that we observed for *atoh1a* and *deltaA*. Curiously, we did not observe an increase in the extent of the *etv4* domain, which is a direct transcriptional target of Fgf signaling. However, the actual levels of Etv4 could be increased, which would be hard to evaluate by the conventional *in situ* approach. On the other hand, Mcf2lb may play an additional role in the pLLP, aside from regulating RhoA activity. In summary, we can conclude that, although the overall process of NM deposition is probably not disrupted, a formation of unstable rosettes with a compromised microlumen leads to the deposition of large or small clusters of cells at uneven intervals.

### Is Mcf2lb involved in the formation or the maintenance of rosettes in the pLLP?

Our study revealed that Mcf2lb regulates apical constriction and rosette integrity. Can we distinguish whether Mcf2lb is involved in the formation and/or maintenance of rosettes? We would argue for the latter for the following reasons. We observe at least some cells that are properly apically constricted within mutant pLLP. In addition, our data showed that even in WT cells, the width of the apical region in constricted cells fluctuates. This implies that this region is under tension and that there is an active mechanism in place to maintain apical constriction. We believe this mechanism is, at least in part, mediated by Mcf2lb, because we observe a much wider range of apical region fluctuation in mutant cells compared with WT. Notably, mutant cells are still able to apically constrict to a certain extent and form rosettes of various sizes. This indicates that there is additional signaling to ensure proper apical constriction. In summary, our data argue for a role for Mcf2lb in rosette maintenance, which is ultimately necessary for the formation of a functional sensory organ. In addition, our scRNA-seq dataset provides a wealth of information to look for additional GEFs and GAPs that regulate apical constriction.

## MATERIALS AND METHODS

### Zebrafish husbandry and strains

Adult zebrafish were maintained under standard conditions (Westerfield, 2000). Membranes of the cells in the pLLP were visualized using Tg(*-8.0claudinB:lynGFP*)*^zf106^* and Tg(*prim:lyn2-mCherry*) (Haas and Gilmour, 2006). Tg(*prim:lyn2-mCherry*) is a fortuitous integration of the memRFP, driven by a 3 kb *sox10* promoter that, in addition to the neural crest, also labels the pLLP (Wang et al., 2018). TgBAC(*cxcr4b:F-tractin-mCherry*) was used to label the pLLP in red fluorescence for scRNA-seq (Yamaguchi et al., 2022). TgBAC(*cxcr4b: LexPr,cryaa:GFP)* was used in combination with the plasmid pDest-Cg2-LexOP-secGFP to mosaically induce expression of sec-GFP in the pLLP (Durdu et al., 2014). TgBAC (*cxcr4b:AHPH-GFP*) was used to examine RhoA activity within the pLLP (Qian et al., 2023 preprint).

### Embryo dissociation and FACS

We collected 30 hpf Tg(*-8.0claudinB:lynGFP*)*^zf106^*/TgBAC(*cxcr4b:F-tractin-mCherry*) zebrafish embryos. These were euthanized in 1.7 ml microcentrifuge tubes. Embryos were deyolked using a calcium-free Ringer's solution (116 mM NaCl, 2.6 mM KCl, 5 mM HEPES pH 7.0) by gently pipetting up and down with a P200 pipet. Embryos were incubated for 5 min in Ringer's solution. Embryos were transferred to pre-warmed protease solutions (0.25% trypsin, 1 mM EDTA, pH 8.0, PBS) and collagenase P/HBSS (100 mg/ml) was added. Embryos were incubated at 28°C for 15 min and were homogenized every 5 min using a P1000 pipet. The Stop solution (6×, 30% calf serum, 6 mM CaCl_2_, PBS) was added and samples were centrifuged (350 ***g***, 4°C for 5 min). Supernatant was removed and 1 ml of chilled suspension solution was added (1% fetal bovine serum, 0.8 mM CaCl_2_, 50 U/ml penicillin, 0.05 mg/ml streptomycin, DMEM). Samples were centrifuged again (350 ***g***, 4°C for 5 min) and supernatant was removed. Then 700 μl of chilled suspension solution was added and cells were resuspended by pipetting. Cells were passed through a 40 μm cell strainer into a FACS tube and kept on ice. GFP- and RFP-positive cells were sorted using FACS on a BD Symphony cell sorter into sorting buffer (50 μl PBS/2% bovine serum albumin) in a siliconized 1.5 ml tube.

### scRNA-seq library construction

FAC-sorted live cells were used for scRNA-seq. Approximately 15,000 cells were loaded into the Chromium Single Cell Controller to generate barcoded RT products (Chemistry Ver 3.0; 10x Genomics). The 10x Genomics chromium scRNA-seq library was sequenced using Illumina NovaSeq 6000 to a depth of at least 25,000 reads per cell.

### Quality control and unsupervised clustering

Single cell reads were aligned to the Ensembl version GRCz11 of the zebrafish genome by the Integrated Genomics Laboratory (Oregon Health & Science University) using Cell Ranger (version 3.1.0; 10x Genomics). UMI count matrix was analyzed using Seurat (version 4.0.1) (Butler et al., 2018). Quality control filtered out genes expressed in fewer than three cells, cells with less than 1900 unique genes and cells that expressed greater than 5% of mitochondrial transcripts. The remaining 3851 cells were subjected to further analysis. Linear dimensionality reduction, clustering and UMAP visualization were performed with Seurat (Butler et al., 2018). Briefly, Principal Component Analysis was performed to project the 2000 most variable genes into 20 principal components. Twenty-two clusters were identified with the Seurat implementation of the Louvain Algorithm using a resolution of 0.8, and visualized with two UMAP dimensions. Clusters were manually annotated using a whole zebrafish single-cell transcriptome atlas (Farnsworth et al., 2020) as well the zebrafish database of gene expression (ZFIN expression atlas: Thisse et al., 2001). Notably, a cluster optimization algorithm (https://bioconductor.org/packages/release/bioc/html/bluster.html) identified the resolution 0.7 as optimal. However in that case, *msx1b*-positive fin epidermal cells co-clustered with pLLP cells; these populations separated into distinct clusters at the resolution 0.8, which we used for further analysis. A subset of pLLP clusters was identified using literature-derived markers and then subclustered to identify unique pLLP transcriptomic states (Farnsworth et al., 2020). pLLP cells subclustered into three groups using the resolution 0.2. However, cluster optimization identified the resolution 0.3 as optimal, leading to four clusters. This was a result of the follower cells separating into two very similar populations (Fig. S7). As such, we used the resolution 0.2 for further analysis.

### Gene Ontology analysis

GO analysis was performed using the R package GO.db (https://bioconductor.org/packages//2.7/data/annotation/html/GO.db.html), biomaRt (Durinck et al., 2005), clusterProfiler (Yu et al., 2012) and org.Dr.eg.db (https://bioconductor.org/packages/release/data/annotation/html/org.Dr.eg.db.html). KEGG pathway analysis was performed using the DAVID online platform (Dennis et al., 2003; Hosack et al., 2003).

### *In situ* hybridization and whole mount immunostaining

RNA *in situ* hybridization was performed as described previously (Andermann et al., 2002). Digoxygenin-labeled antisense RNA probes were generated for the following genes: *mcf2lb*, *twf2b*, *arhgef4*, *fhdc1*, *atoh1a* (Itoh and Chitnis, 2001) and *deltaA* (Itoh and Chitnis, 2001).

We collected 45 hpf embryos and fixed them in BT fixative (anti-ZO-1), glyofixx (Thermo Fisher Scientific, anti-Rock2a) or Bouin's fixative (Polysciences; anti-pMRLC) (Westerfield, 2000) overnight at 4°C. After removing the fixative, embryos were washed with PBS/0.1% Triton washes, embryos were blocked with phosphate-buffered saline with 0.1% Triton (PBTx)/5% goat serum/1% bovine serum albumin, 1% DMSO before incubating in primary antibody at 4°C overnight. The embryos were washed in PBS/0.1% Triton and incubated in secondary antibody at 4°C overnight and then washed with PBS/0.1% Triton. After the final wash, embryos were mounted in 50% PBS/50% glycerol for imaging. Primary antibodies were used at the following dilutions: mouse anti-ZO-1 (1:500), rabbit anti-Rock2a (1:50) and rabbit anti-pMRLC (1:20) (see Table S1). Secondary antibodies (used at 1:750 concentration) were goat anti-rabbit Alexa Fluor 568, goat anti-mouse Alexa Fluor 568 and goat anti-chicken Alexa Fluor 488 (Thermo Fisher Scientific). Nuclei were visualized with DAPI.

### CRISPR-Cas9-mediated knockout

Three single guide RNAs targeting exons 6, 10 and 21 for CRISPR-Cas9 targeting of *mcf2lb* were designed and injected as previously described (Shah et al., 2015) (see Table S1). All three guides were injected together into Tg(*-8.0claudinB:lynGFP*)*^zf106^*-positive embryos. At 3 dpf, embryos were screened for a pLL formation phenotype and genotyped to assess CRISPR efficiency. F0-injected embryos were raised to adulthood, in-crossed, and F1 embryos were screened at 3 dpf for a pLL formation phenotype. Positive F0 adults were out-crossed to a WT background, and progeny were raised to adulthood. A stable line was identified when F1 adults were in-crossed and their subsequent progeny were screened at 3 dpf for the pLL phenotype, which was observed with Mendelian inheritance.

All three guides were efficient in their cutting and produced indels. We generated two mutant alleles that contained mutations in three distinct exons (Fig. S3A). The first allele, nl25 (allele 1), contained an 8 bp insertion in exon 6 which produces an early STOP within the insertion; a 6 bp deletion and an 18 bp insertion in exon 10 (a net 12 bp insertion) that also ultimately leads to an early STOP. The second allele, nl26 (allele 2), contained a 7 bp insertion in exon 6 and results in an early STOP shortly after the insertion; a 442 bp deletion and 18 bp insertion that produces a net 424 bp deletion. In addition, both alleles contained a 7 bp deletion in exon 21 (early STOP shortly after the deletion) (Fig. S3A). The *mcf2lb* mutant population used in this study contains a mixed population of both nl25 and nl26. We observed no phenotypic differences between the two alleles (Fig. S3B).

To distinguish between nl25 and nl26, regions including exon 6, exon 10 and exon 21 were PCR amplified from adult genomic DNA and digested with restriction enzymes. Phusion High-Fidelity DNA Polymerase (Thermo Fisher Scientific) was used for exon 6, and Taq DNA Polymerase (New England Biolabs) was used for exons 10 and 21. Standard PCR conditions according to the manufacturers’ instructions were used. Annealing temperatures of 63°C, 62°C and 55°C were used for exon 6, exon 10 and exon 21 PCR amplification, respectively. PCR-amplified DNA was digested with MwoI and HpyCH4III in separate reactions for exon 6, DrdI for exon 10, and Cac8I for exon 21. Amplification of the region of DNA containing exon 10 in nl26 proved to be challenging.

### Plasmids, injections and LexPr/LexOP induction

Sp6-Par3-tagRFP plasmid was used from Harding and Nechiporuk (2012). *par3-tagRFP* mRNA was synthesized using the mMessage mMachine Kit (Life Technologies) and microinjected at 250 pg/embryo. pDEST-Cg2-LexOP-secGFP (Wang et al., 2018) was microinjected into TgBAC*(cxcr4b:LexPr,cryaaGFP)* transgenic embryos (Durdu et al., 2014) at 5 pg/embryo. Expression of LexOP-secGFP was induced by treating 29 hpf TgBAC(*cxcr4b:LexPr; cryaa:GFP*) transgenic embryos with 20 μM mifepristone (RU486, Sigma-Aldrich) for 6 h at 31°C.

### Transplantation experiments and time-lapse live imaging

Transplantation experiments were carried out as previously described (Nechiporuk and Raible, 2008). All host embryos expressed the Tg(*-8.0claudinB:lynGFP*)*^zf106^* transgene and were either in a WT or *mcf2lb* mutant background. Donor cells were derived from WT or *mcf2lb* mutant Tg(*prim:lyn2-mCherry*) transgenic embryos. Embryos were screened at ∼28 hpf and then mounted at ∼30 hpf for live imaging.

For time-lapse imaging, embryos were anesthetized in 0.02% tricaine (MS-222; Sigma-Aldrich) embedded in 1.2% low-melting point agarose and imaged either using a 20×/NA=0.95 water dipping lens or a 40×/NA=1.25 silicone lens on an upright Fluoview 3000 confocal microscope (Olympus). For overnight time-lapse imaging, pLLPs were imaged between 30 and 44 hpf. For high-resolution pLLP imaging and imaging of Par-3-tagRFP in the migrating pLLP, pLLPs were imaged at ∼30 hpf.

### Foci versus points distinction

Gatherings of membranes were depicted as either foci or points. To determine what qualifies as a focus versus a point, the width and height of each gathering of membrane in WT pLLPs was measured. These values were averaged and a gathering of membrane was determined as a focus if its value landed between one standard deviation above or below the mean of the width and the height of all WT foci. If a gathering of membrane did not meet these criteria, it was determined to be a point.

### Apical constriction quantification

Using the Imaris Cells function, all cells in the pLLP were 3D reconstructed. In the trailing 70% of the pLLP, the ACI of all cells was measured. ACIs are the ratio between the apical width of the cell, 1 μm below the apical top of the cell, and the basal width, 1 μm above the basal bottom of the cell. Cells were categorized as cells incorporated into rosettes if they touched or reached towards a focus or point. Cells were categorized as touching multiple points if cells contacted multiple foci or points. Cells were not included in analysis if they were dividing, did not span the entire length of the pLLP or were considered sheath cells, those cells that lie entirely flat along the top of the pLLP, closest to the skin. All other cells were categorized as not being incorporated into rosettes.

### Membrane variability and secGFP quantification

The apical width 1 μm below the top of the transplanted cell was measured throughout a 1-h imaging period. Membrane variability was defined as the standard deviation of the apical membrane width of a cell over the course of this imaging period.

Normalized fluorescence of secGFP was measured by dividing the total fluorescence of GFP in the microlumen by the total fluorescence of cells expressing secGFP that contribute to the rosette centers of the trailing-most rosette of pLLPs and deposited NM2. The same circular region of interest (ROI) was used to measure the fluorescence in the presumptive microlumen across all samples.

### Polarity marker, RhoA downstream signaling components and RhoA sensor quantification

For ZO-1 signal quantification, we measured the fluorescence intensity of an ROI for the top half (apical) and bottom half (basal) parts of the pLLP. For Par-3, we measured the apical fluorescence intensity defined as half of the pLLP width and height. These ROI values were then divided by total fluorescent intensity values throughout the entire pLLP to achieve the percentage of fluorescence intensity that was apically or basally localized (ZO-1) or the percentage of fluorescence intensity that was apically localized (Par-3).

For ZO-1 signal in NMs, we measured the fluorescence intensity in an ROI that was the top half (apical) and bottom half of the NM (basal). The ROI values were then divided by the total NM fluorescence intensity to calculate the percentage of apically and basally localized signal.

For Rock2a and pMRLC quantification, we first obtained fluorescent intensity values throughout the entire pLLP and then normalized these measurements to the number of the cells within the pLLP (Fig. 9C,G). To obtain the level of Rock2a or pMRLC that was localized to gatherings of membranes, we measured the fluorescence intensity in a consistent ROI that included the rosette centers. The ROI dimensions were determined by using the mean width and length of a focus in WT embryos and one-third of the mean depth of the pLLP. The ROI dimensions were determined separately for each experiment. The fluorescent intensity measurements at gatherings of membranes were not normalized and are presented as individual values.

RhoA sensor fluorescent intensity values were obtained by measuring the fluorescence of the RhoA sensor in a circular ROI (1 µm in diameter) at the presumptive rosette centers and subtracting the background signal from that value. The ROI was consistent in size across pLLPs used in this analysis.

### Image processing

Fig. 3 full body 3 dpf embryo images were composited from images of individual body segments (head, trunk, tail) that were stitched together using the auto-blend feature on Photoshop. Images were processed using ImageJ (Abramoff et al., 2004; Schindelin et al., 2012), Imaris (Bitplane) or Photoshop (Adobe) software.

### Statistics

Data were analyzed in PRISM and R. Before statistical analysis, data were tested for normal distributions using normality tests and Q-Q plots. We analyzed parametric data using two-tailed unpaired *t*-tests (Figs 3, 4, 5H, 7-9; Figs S4G,J, S5G,I, S6). To analyze data that were not parametric, we used Mann–Whitney *U*-tests assuming equal variances (Figs 5G,I,J, 6; Figs S4H,I, S5H) or a Kruskal–Wallis test (Fig. S3). We used a chi-square test to analyze categorical variables (Fig. 5F). *P*-values are indicated as follows: *P<0.05, **P<0.01, ***P<0.001.

## Supplementary Material

10.1242/develop.201898_sup1 Supplementary information
