# Supplementary material for: RhoA GEF Mcf2lb regulates rosette integrity during collective cell migration
Source: Development. 2024 Jan 2;151(1):dev201898. doi: 10.1242/dev.201898 (PMC10820872; doi:10.1242/dev.201898)
Supplement: Supplementary information [file develop-151-201898-s1.pdf]

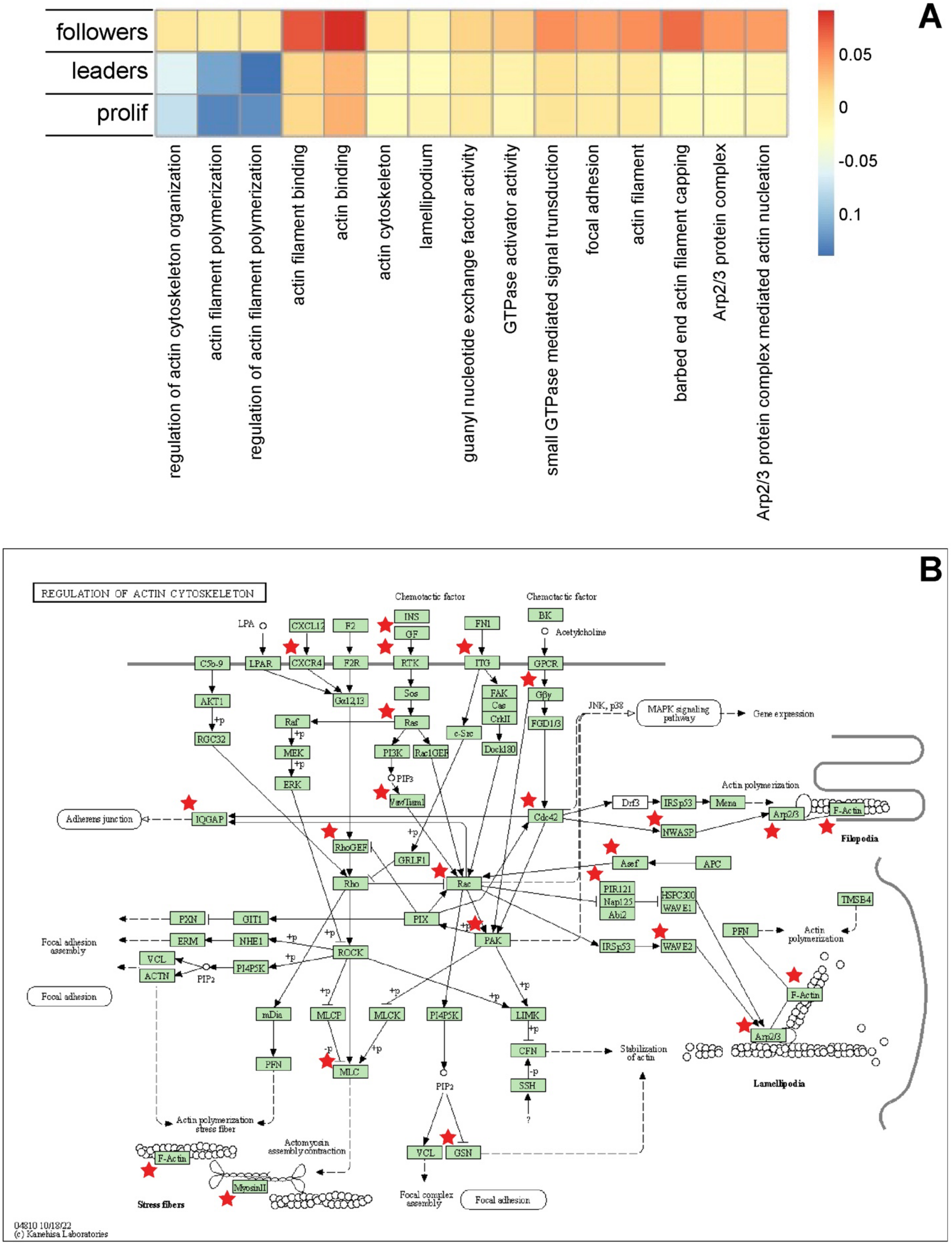

Development • Supplementary information

Fig. S1. GO term and KEGG analysis of the scRNA-seq data set for genes that regulate actin dynamics.

(A) Heatmap illustrating pLLP module scores for GO terms that are associated with various processes involving actin. (B) Regulation of actin cytoskeleton KEGG pathway map shows presence (red star) of molecules that are associated with of this process in our scRNA-seq data set.

Development • Supplementary information

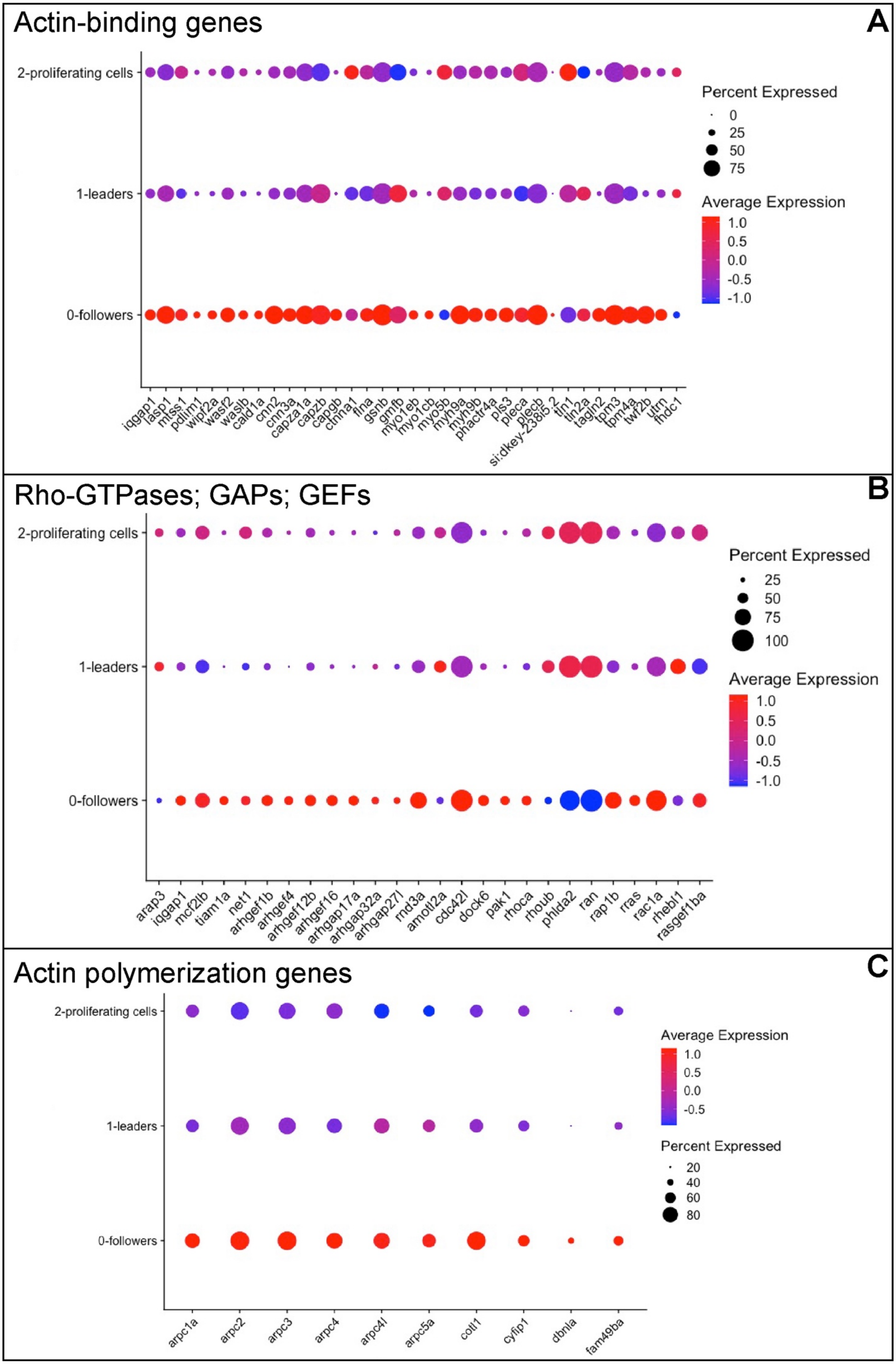

Development • Supplementary information

Fig. S2. Expression of genes that regulate actin dynamics in pLLP clusters. (A-C) Using our scRNA-seq data set, we performed GO term enrichment analysis to reveal expression of actin-binding genes (A), Rho GTPases, GAPS, and GEFs (B), and actin polymerization genes (C) among the three pLLP specific clusters. Note enhancement of expression of actin-binding genes and actin polymerization genes in the follower population compared to the leaders and proliferating cells.

Development • Supplementary information

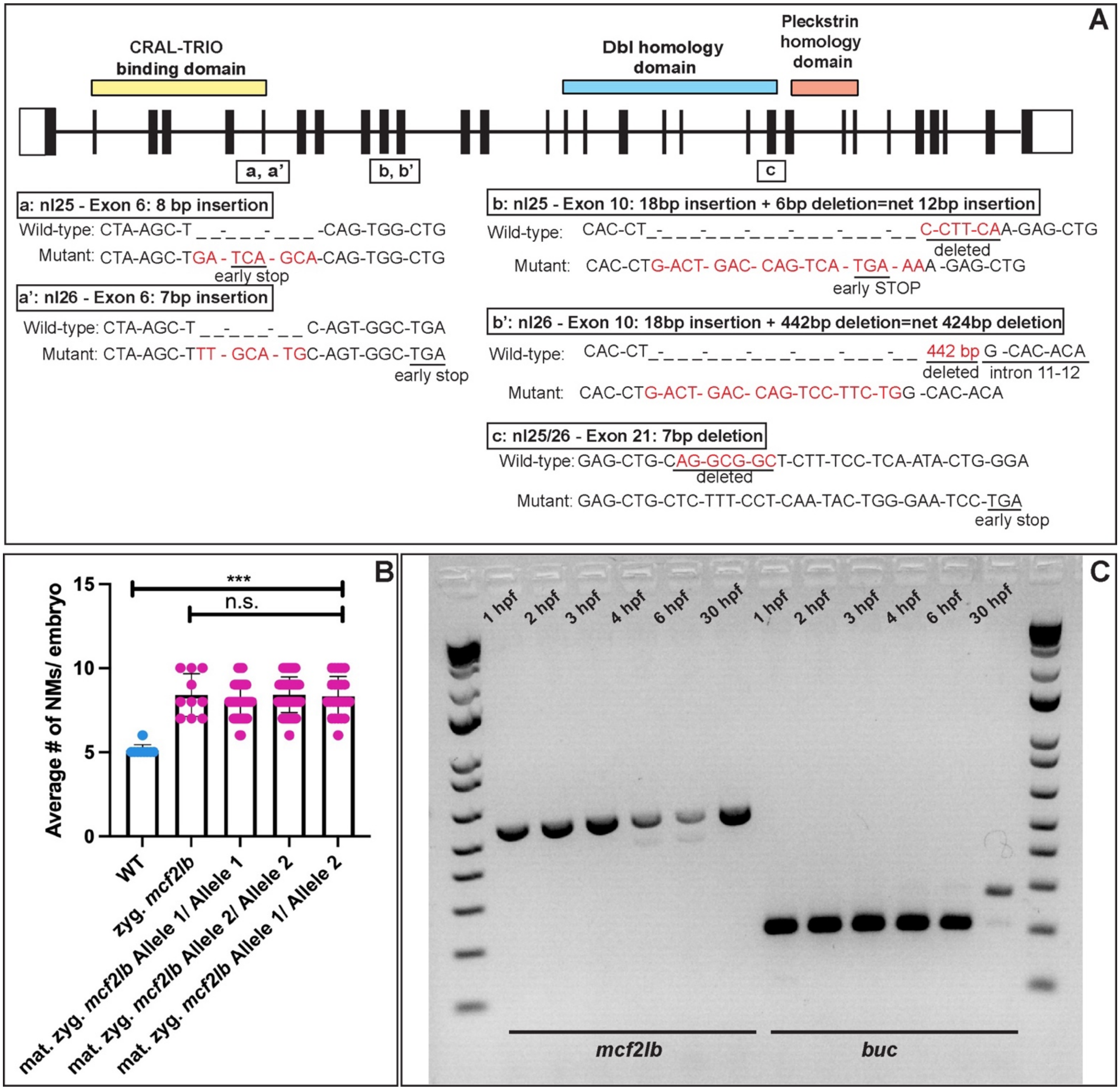

Fig. S3. *mcf2lb* mutant validation.

(A) Schematic and sequence of the four different CRISPR/Cas9-induced mutations. There are 2 mutations in exon 6, 2 mutations in exon 10, and 1 mutation in exon 21. This resulted in two alleles further designated as nl25 (Allele 1), nl26 (Allele 2). All mutations lead to early STOPs as indicated. (B) Phenotypic comparison between WT (n = 9 embryos), zygotic *mcf2lb* mutant embryos (n = 10 embryos), maternal zygotic *mcf2lb* Allele 1/ Allele 1 (n = 56 embryos), maternal zygotic *mcf2lb* Allele 2/ Allele 2 (n = 60 embryos), maternal zygotic *mcf2lb* Allele 1/ Allele 2 (n = 24 embryos). Note there is no significant differences in phenotype between the two alleles. Error bars are SD. *** = $p < 0.001$ (Kruskal – Wallis test). (C) Expression of *mcf2lb* and *buc* during early development 1 – 6 hpf and at 30 hpf.

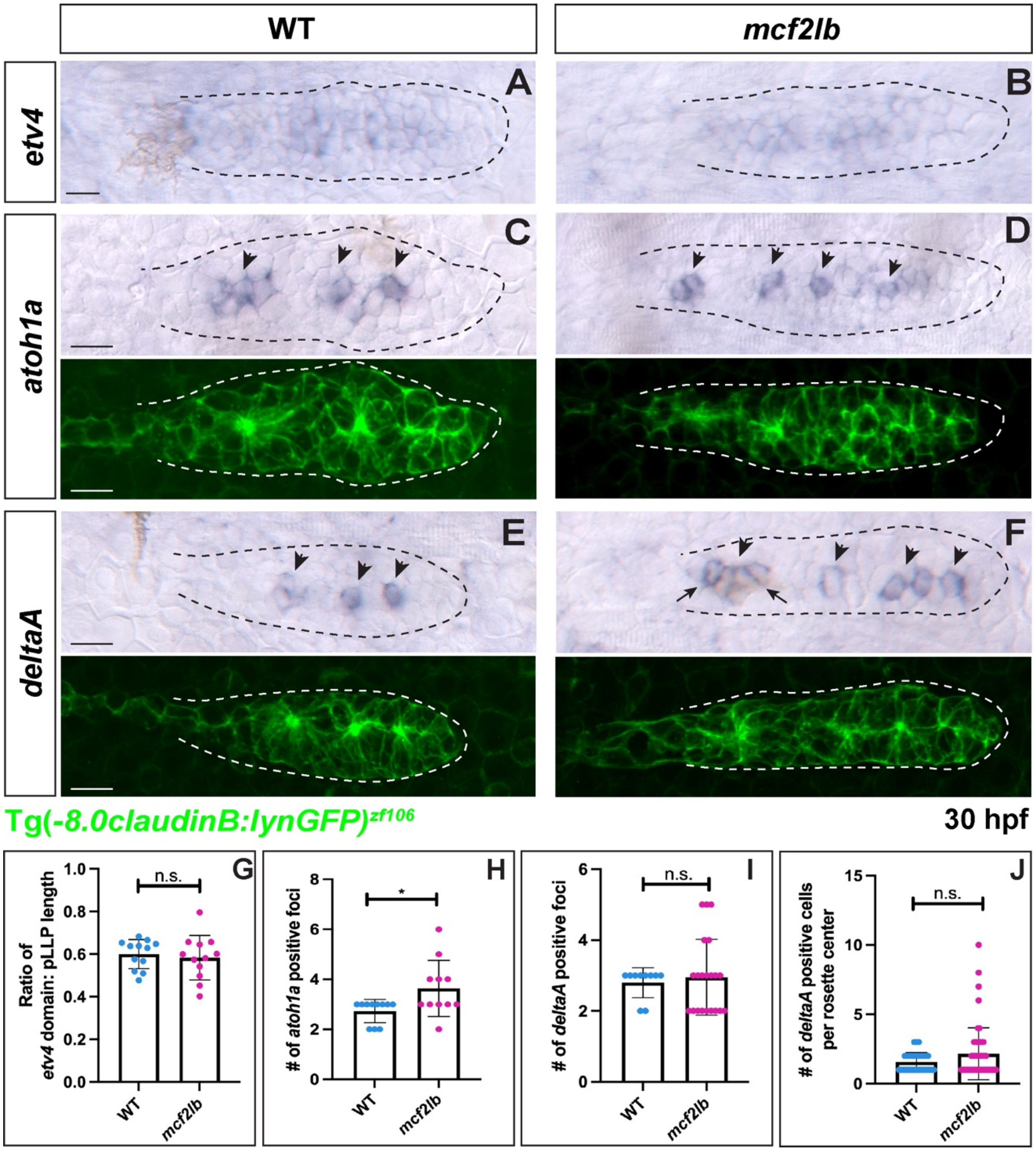

Fig. S4. Hair cell specification during pLLP migration.

(A, B) In situ hybridization of the Fgf signaling component *etv4* at 30hpf. (C – F) In situ hybridization of the hair cell markers *atoh1a* (A, B) and *deltaA* (C, D) in WT and *mcf2lb* mutant pLLs at 30 hpf. Dotted lines indicate pLLP. Arrowheads indicate foci. Arrows indicate foci that contain more than 2 cells expressing *deltaA*. (G) Ratio of *etv4* domain to the length of the pLLP in WT (n = 12 pLLPs) and *mcf2lb* mutant embryos (n = 12 pLLPs). (H) Average number of *atoh1a* positive cells within the pLLP in WT (n = 12 pLLPs) and *mcf2lb* mutants (n = 12 pLLPs). (I) Average number of *deltaA* positive cells within the pLLP in WT (n = 11 pLLPs) and *mcf2lb* mutant embryos (n = 11 pLLPs). (J) Average number of *deltaA* positive cells within the pLLP in WT (n = 30 foci from 10 embryos) and *mcf2lb* mutants (n = 53 foci from 21 embryos). * = $p < 0.05$ (G, J unpaired two tailed *t*-test; H, I Mann – Whitney *U* test). Error bars are SD. Scale bars = 10 μm.

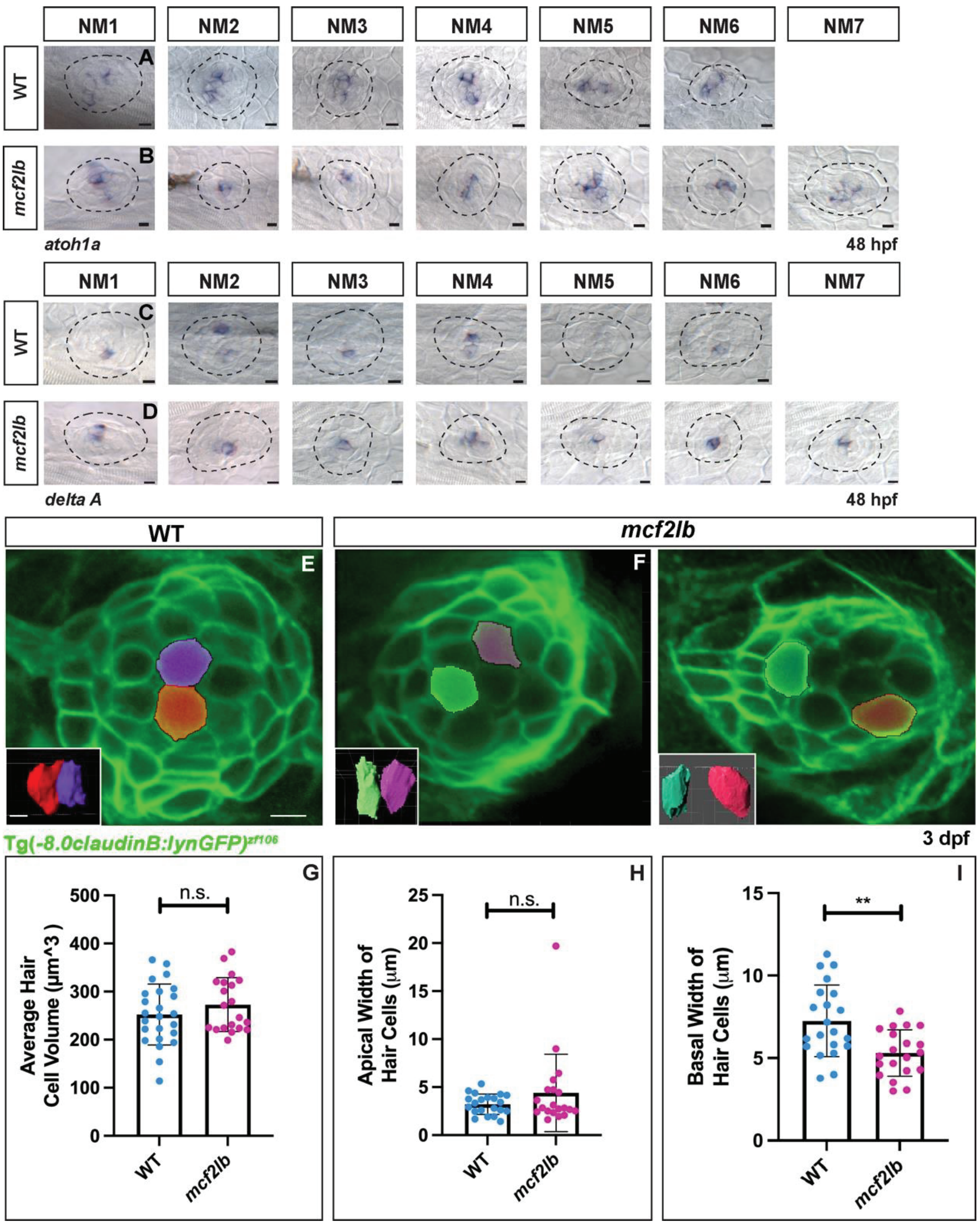

Fig. S5. Hair cells are properly specified in the pLL of *mcf2lb* mutant embryos. (A – D) In situ hybridization of the hair cell markers *atoh1a* (A, B) and *deltaA* (C, D) in WT and *mcf2lb* mutant pLLs at 48 hpf. Dotted lines indicate NM. (E, F) 3D reconstruction of hair cells in NM L5 in WT and (F) *mcf2lb* mutants at 3dpf. (G) Quantification of hair cell volume in WT (n = 24 hair cells from 12 NMs) and *mcf2lb* mutants (n = 20 hair cells from 10 NMs). (H) Quantification of apical width of hair cells in WT (n = 21 hair cells from 10NMs) and *mcf2lb* mutants (n = 20 hair cells from 10NMs). (I) Quantification of basal width of hair cells in WT (n = 21 hair cells from 10NMs) and *mcf2lb* mutants (n = 20 hair cells from 10NMs). ** = $p < 0.01$ (G, I unpaired two tailed *t*-test; H Mann – Whitney *U* test) Error bars are SD. A-D: Scale bars = 5 µm. E, F: Scale bars = 5 µm.

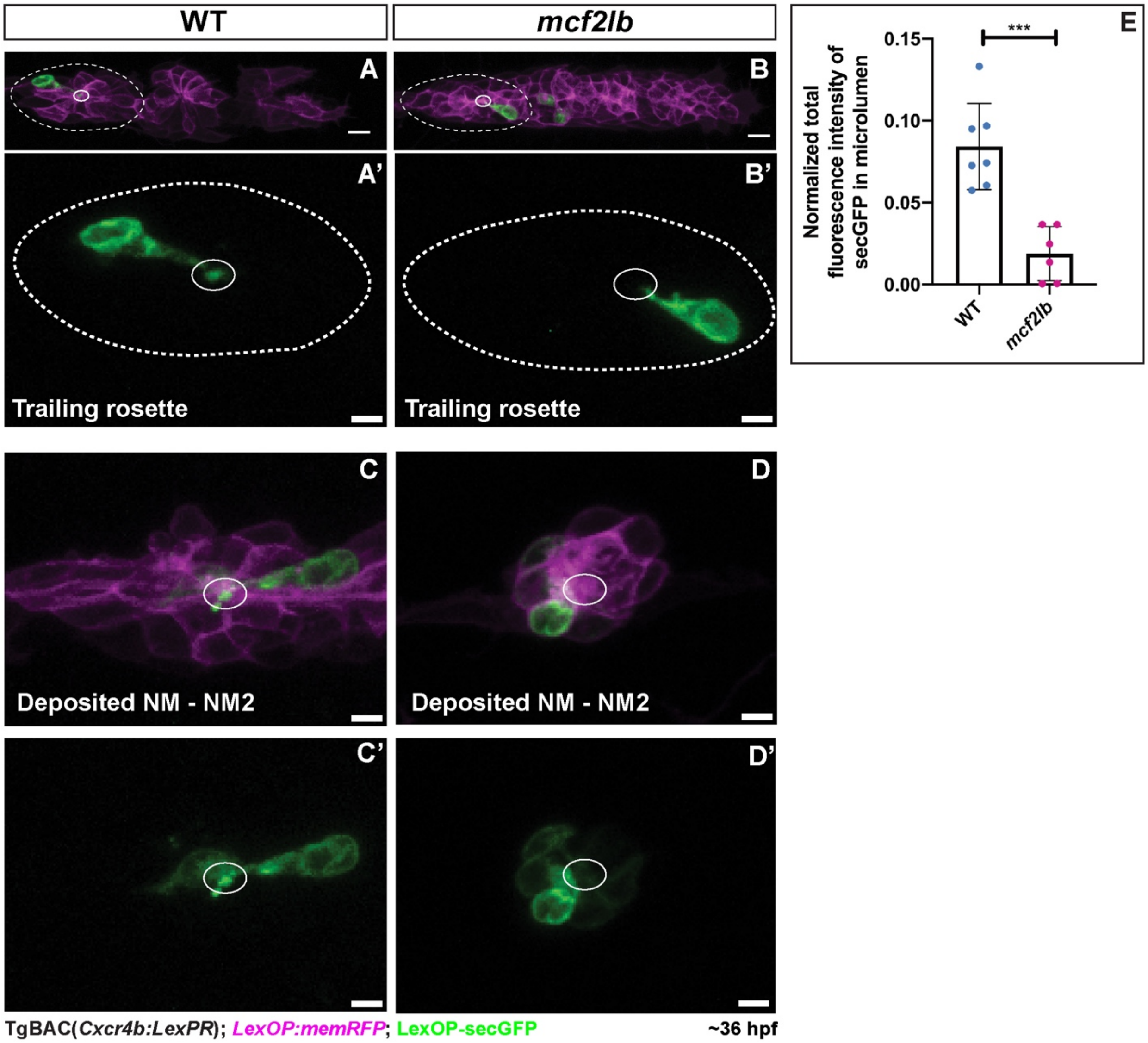

Fig. S6. Microlumen integrity is compromised in the *mcf2lb* mutant pLLP and differentiating NMs.

LexOP-secGFP plasmid was injected into TgBAC(*Cxcr4b:LePR; LexOP:memRFP*) transgenic fish. LexPR was induced for 6 hours (29-35 hpf) to induce expression of secGFP (green) and memRFP (magenta). (A, B) secGFP expression in the trailing rosette of WT and *mcf2lb* mutant pLLPs. Solid ovals denote presumptive microlumen and dotted line marks the trailing rosette. (A', B') Higher magnification of the rosettes outlined in panels A and B. (C, D) secGFP expression in deposited NM2 of WT and *mcf2lb* mutant embryos (presumptive microlumen is outlined). (E) Quantification of fluorescence intensity of secGFP in the microlumen normalized to the fluorescence intensity of cells expressing secGFP that contribute to the rosette centers of trailing rosettes and deposited NM2 in WT (n = 7 microlumen from 7 embryos) and *mcf2lb* mutants (n = 6 microlumen from 5 embryos).*** $p < 0.001$ (unpaired two tailed *t*-test). Error bars are SD. Scale bars in panels A, B= 10 µm and in panels A', B', C, C', D, and D' = 5 µm.

Development • Supplementary information

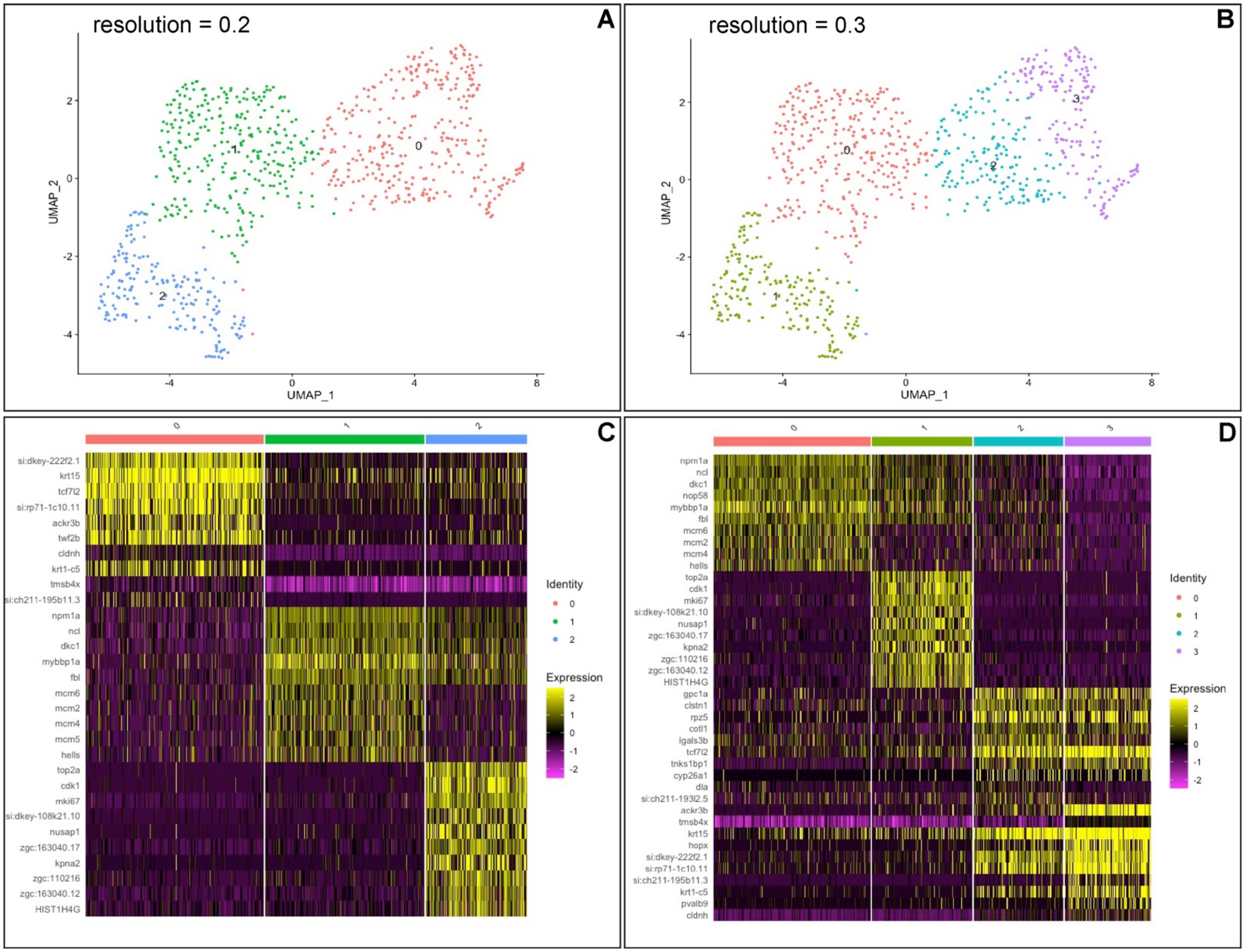

Fig. S7. Subclustering of pLLP cells at different resolutions.

A resolution sweep from 0.1 to 1 at 0.1 increments identified resolution 0.3 as optimally maximizing silhouette width. However, we noted that at this resolution, follower cells (cluster 0 in A) separated into two clusters that have extremely similar gene signatures (cluster 2 and 3 in B): compare gene signature for clusters 2 and 3 (D). This gene signature also contained 9 out of 10 genes from cluster 0 (C).

Development • Supplementary information

Table S1. Key Resources Table

Reagent or resource	Source	Identifier
Primary Antibodies		
Monoclonal Mouse anti ZO-1	Invitrogen	CAT# 33-9100
Rabbit anti Rock-2a	Anaspec	N/A
Polyclonal Rabbit anti pMRLC	Cell Signaling Technology	CAT# 3671
Experimental Models: Organisms/Strains		
Zebrafish: Wildtype-AB strain		
Zebrafish: Tg(-8.0*claudinB: lynGFP)*^{zf106}	(Haas and Gilmour, 2006)	N/A
Zebrafish: Tg(*prim:lyn2-mCherry*)	(Wang et al., 2018)	N/A
Zebrafish: TgBAC(*cxcr4b:F-tractin-mCherry*)	(Yamaguchi et al., 2022)	N/A
Zebrafish: TgBAC *(cxcr4b: LexPr,cryaa:GFP)*	(Durdu et al., 2014)	N/A
Zebrafish: TgBAC (*cxcr4b:AHPH-GFP)*	(Qian et al., 2023; preprint)	N/A
Oligonucleotides		
CRISPR Guide Exon 6<br>5'aattaatacgactcactataggttatcatgctaagctcaggttttagagctagaaatagc 3'	Designed using Chop chop	https://chopchop.cbu.uib.no/
CRISPR Guide Exon 10<br>5'aattaatacgactcactatagattggtcagctcttgaagggttttagagctagaaatagc 3'	Designed using Chop chop	https://chopchop.cbu.uib.no/
CRISPR Guide Exon 21<br>5'aattaatacgactcactatagggctcagtggagctgcagggttttagagctagaaatagc 3'	Designed using Chop chop	https://chopchop.cbu.uib.no/
mcf2lb in situ hybridization probe<br>Forward: 5'GATGGAGCTCGCCAGGTTTA 3'<br>Reverse:<br>5'CCAAGCTTCTAATACGACTCACTATAGGGAGATCCCTCTCCTCTTCTGGGTC 3'	N/A	N/A
arhgef4 in situ hybridization probe<br>Forward: 5' GCTCAAGTACACCAACCCACA 3'<br>Reverse:<br>5'CCAAGCTTCTAATACGACTCACTATAGGGAGAAACACAAGTTCAGGCACCGAG 3'	N/A	N/A
twf2b in situ hybridization probe<br>Forward: 5' CACAGAGGACGAGCGAAGAAT 3'<br>Reverse: 5'<br>CCAAGCTTCTAATACGACTCACTATAGGGAGAGGCCTTGTAAACCATGCCAG 3'	N/A	N/A
fhdc1 in situ hybridization probe<br>Forward: 5'AAAGCATGCCAGCCAGAAGA 3'<br>Reverse:<br>5'CCAAGCTTCTAATACGACTCACTATAGGGAGATTGCTGAGCATCTAGCAACAC	N/A	N/A
buc positive control maternal contribution PCR<br>Forward: 5' GCAACCTCACCACCCAGTAA 3'<br>Reverse: 5' GCATGGGTGCATGTGGAATC 3'	N/A	N/A

Development • Supplementary information

mcf2lb maternal contribution PCR:<br>Forward: 5' GAAGGAGTCGAGTCCCCTCT 3'<br>Reverse: 5' CACTTGCAGTCTCTGAGCCA 3'	N/A	N/A
atoh1a in situ hybridization probe	(Itoh and Chitnis, 2001)	N/A
deltaA in situ hybridization probe	(Itoh and Chitnis, 2001)	N/A
Recombinant DNA		
Plasmid: Sp6-Par3-tagRFP-STOP	(Gong et al., 2002; Rieger and Sagasti, 2011)	N/A
Plasmid: pDest-Cg2-LexOP-secGFP	(Durdu et al., 2014)	N/A
Software and Algorithms		
Imaris	Bitplane	https://imaris.oxinst.com/
ImageJ	(Schindelin et al., 2012)	
PRISM	GraphPad	GraphPad Software, La Jolla California USA, www.graphpad.com

Supplementary References

Durdu, S., Iskar, M., Revenu, C., Schieber, N., Kunze, A., Bork, P., Schwab, Y. and Gilmour, D. (2014). Luminal signalling links cell communication to tissue architecture during organogenesis. *Nature* **515**, 120-124. doi:10.1038/nature13852

Haas, P. and Gilmour, D. (2006). Chemokine signaling mediates self-organizing tissue migration in the zebrafish lateral line. *Dev. Cell* **10**, 673-680. doi:10.1016/j.devcel.2006.02.019

Itoh, M. and Chitnis, A. B. (2001). Expression of proneural and neurogenic genes in the zebrafish lateral line primordium correlates with selection of hair cell fate in neuromasts. *Mech. Dev.* **102**, 263-266. doi:10.1016/S0925-4773(01)00308-2

Qian, W., Yamaguchi, N., Lis, P., Cammer, M. and Knaut, H. (2023). Pulses of RhoA signaling sitmulate actin polymerization and flow in protrusions to drive collective cell migration. *bioRxiv*. doi:10.1101/2023.10.03.560679v1.

Rieger, S. and Sagasti, A. (2011). Hydrogen peroxide promotes injury-induced peripheral sensory axon regeneration in the zebrafish skin. *PLoS Biol.* **9**, e1000621. doi:10.1371/journal.pbio.1000621

Schindelin, J., Arganda-Carreras, I., Frise, E., Kaynig, V., Longair, M., Pietzsch, T., Preibisch, S., Rueden, C., Saalfeld, S., Schmid, B. et al. (2012). Fiji: an open-source platform for biological-image analysis. *Nat. Methods* **9**, 676-682. doi:10.1038/nmeth.2019

Wang, J., Yin, Y., Lau, S., Sankaran, J., Rothenberg, E., Wohland, T., Meier-Schellersheim, M. and Knaut, H. (2018). Anosmin1 shuttles Fgf to facilitate its diffusion, increase its local concentration, and induce sensory organs. *Dev. Cell* **46**, 751-766.e712. doi:10.1016/j.devcel.2018.07.015

Yamaguchi, N., Zhang, Z., Schneider, T., Wang, B., Panozzo, D. and Knaut, H. (2022). Rear traction forces drive adherent tissue migration in vivo. *Nat. Cell Biol.* **24**, 194-204. doi:10.1038/s41556-022-00844-9

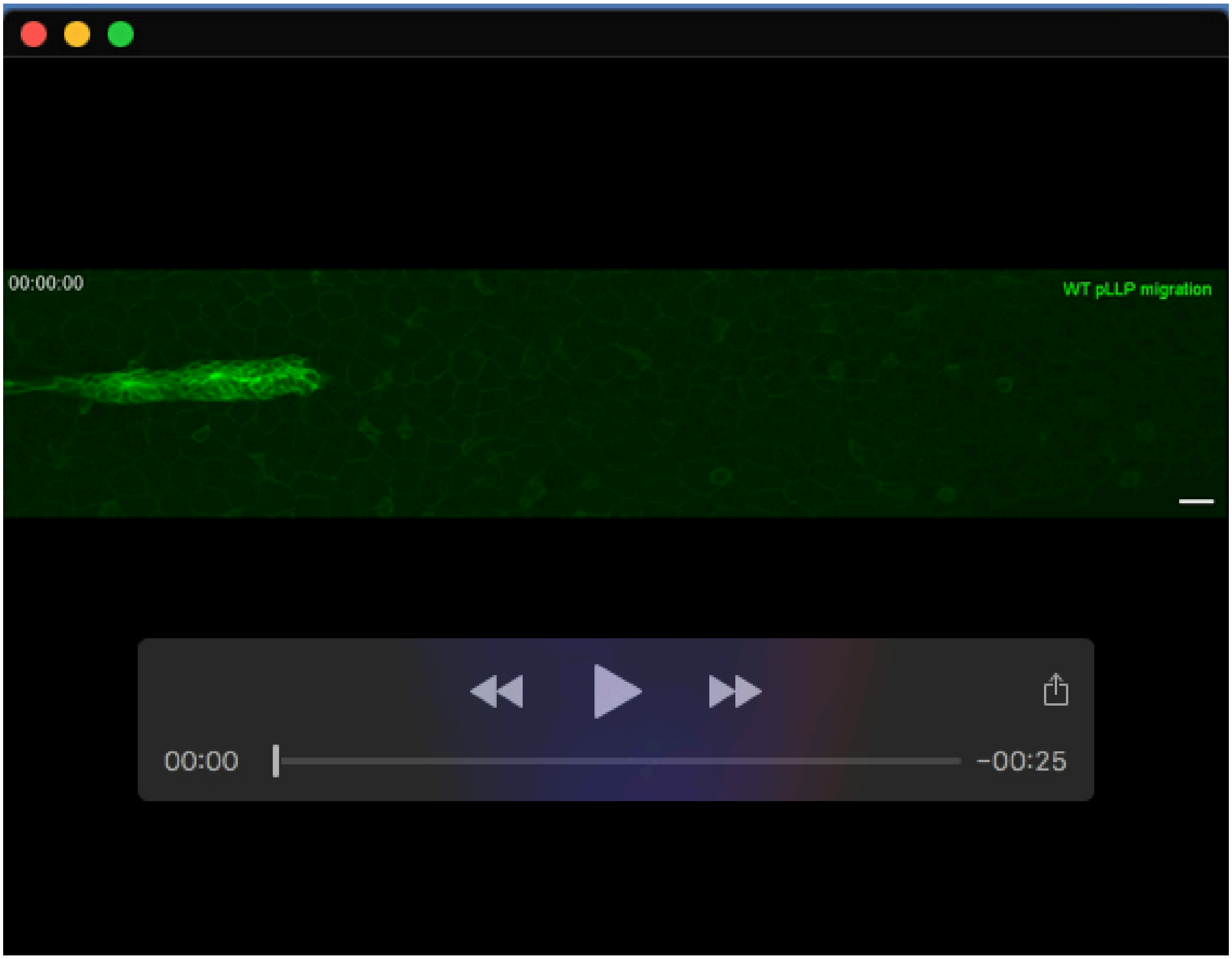

Movie 1. WT pLLP migration and NM deposition. Time lapse confocal projections of Tg(-*8.0claudinB:lynGFP*)^{zf106} expressing cells in a WT embryo. Embryo was imaged continuously starting at 30 hpf for 15 hours. Scale bar = 20 μm.

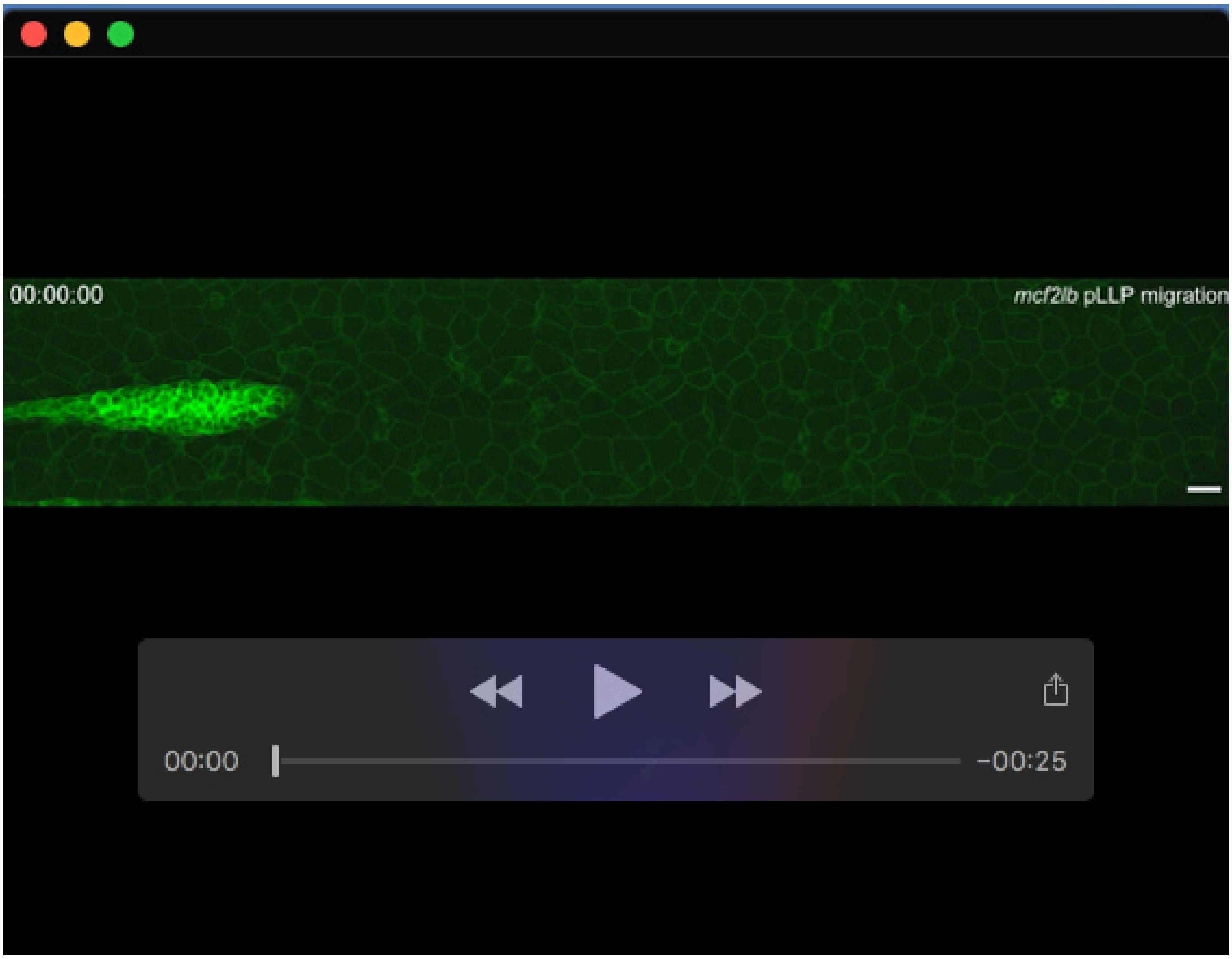

Movie 2. *mcf2lb* mutants show abnormal NM deposition behavior. Time lapse confocal projections of Tg(*-8.0claudinB:lynGFP*)^{zf106} expressing cells in a *mcf2lb* mutant embryo. Embryo was imaged continuously starting at 30 hpf for 14 hours. Scale bar = 20 μm.

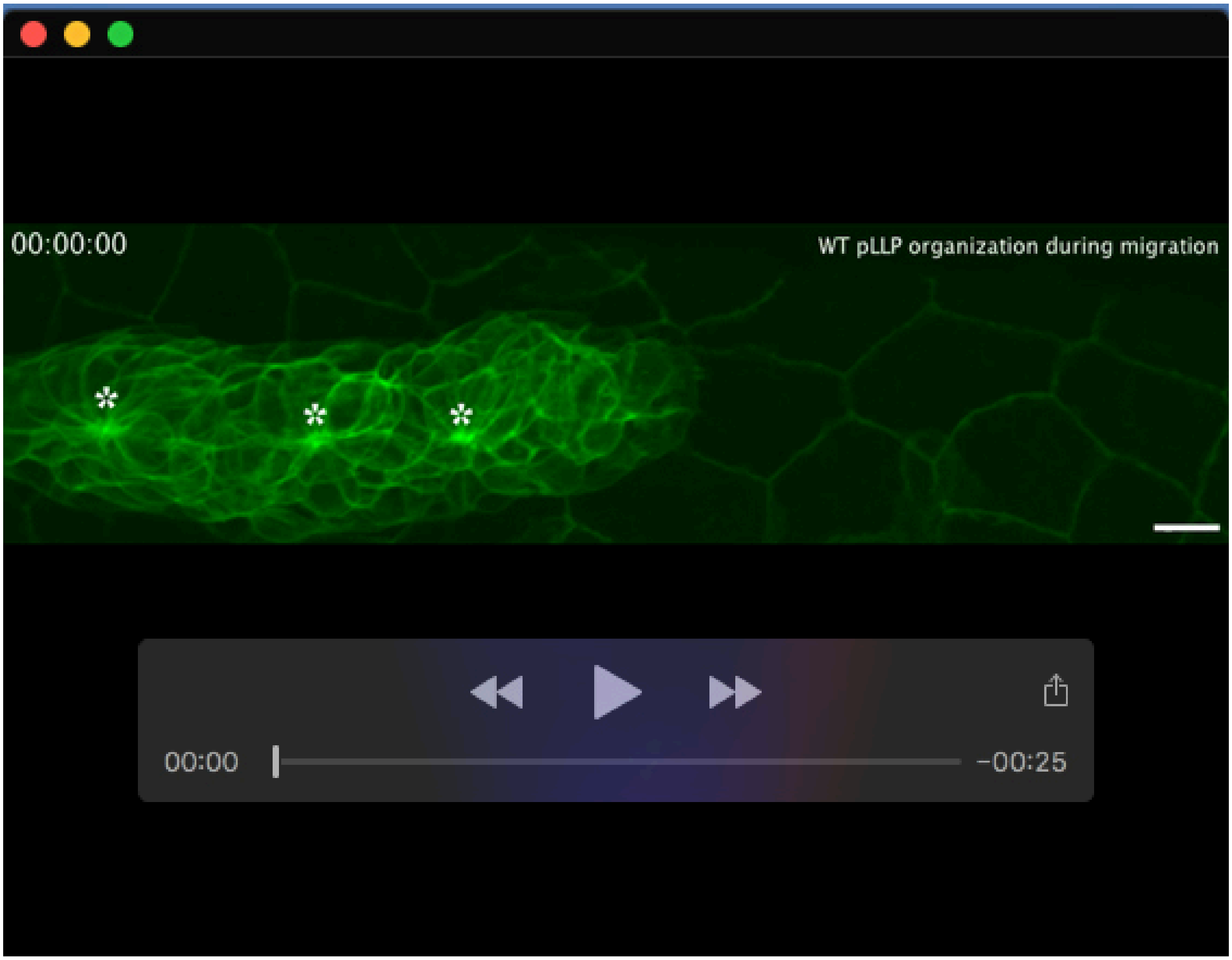

Movie 3. WT pLLP organization during migration. Time lapse confocal projections of Tg(-*8.0claudinB:lynGFP*)^{zf106} expressing cells in a WT embryo at high magnification. Embryo was imaged continuously starting at 30 hpf for 1.5 hours. Scale bar = 10 µm.

Development • Supplementary information

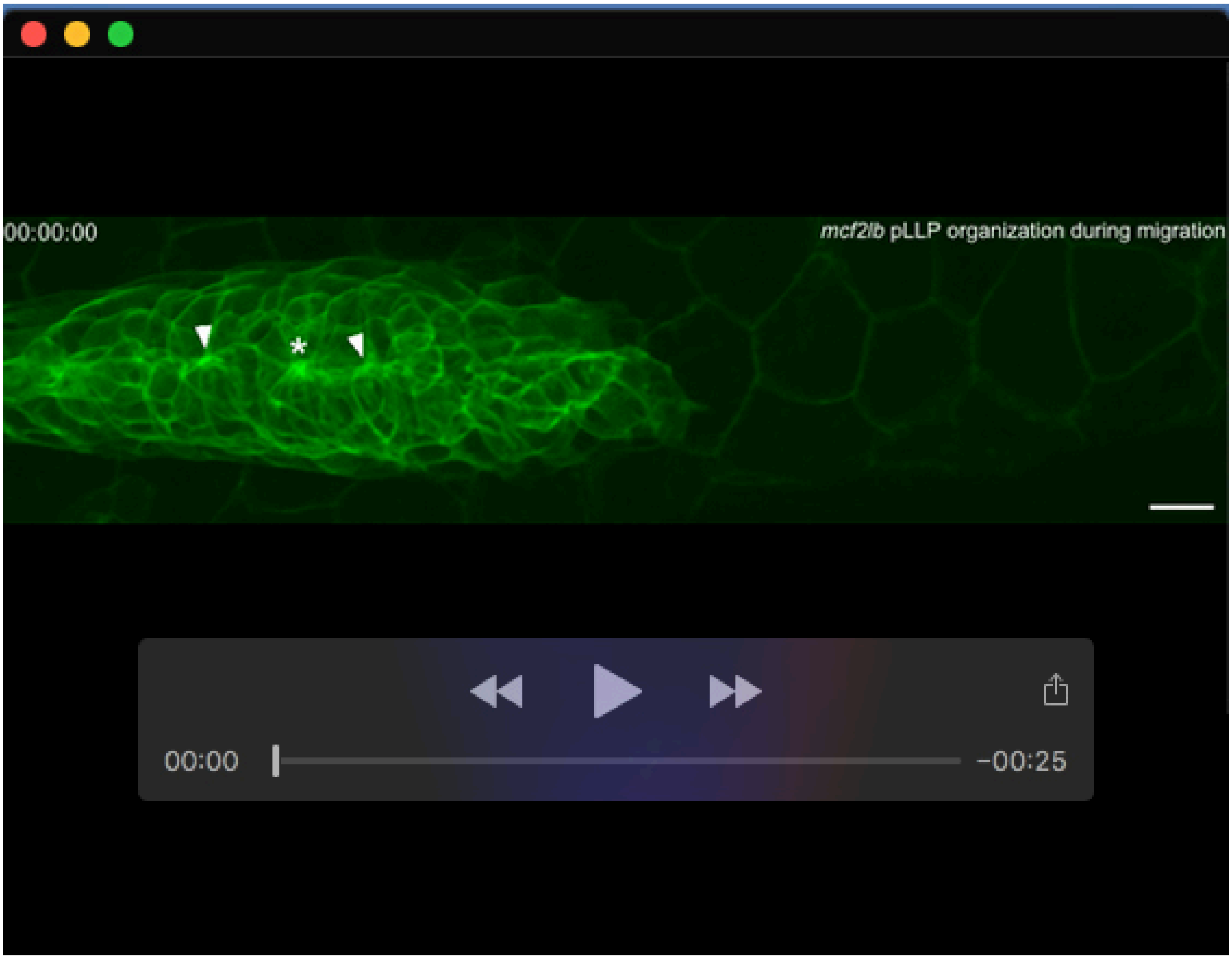

Movie 4. *mcf2lb* mutants show abnormal NM deposition behavior. Time lapse confocal projections of Tg(*-8.0claudinB:lynGFP*)^{zf106} expressing cells in a *mcf2lb* mutant embryo at high magnification. Embryo was imaged continuously starting at 30 hpf for 1.5 hours. Scale bar = 10 μm.

Development • Supplementary information

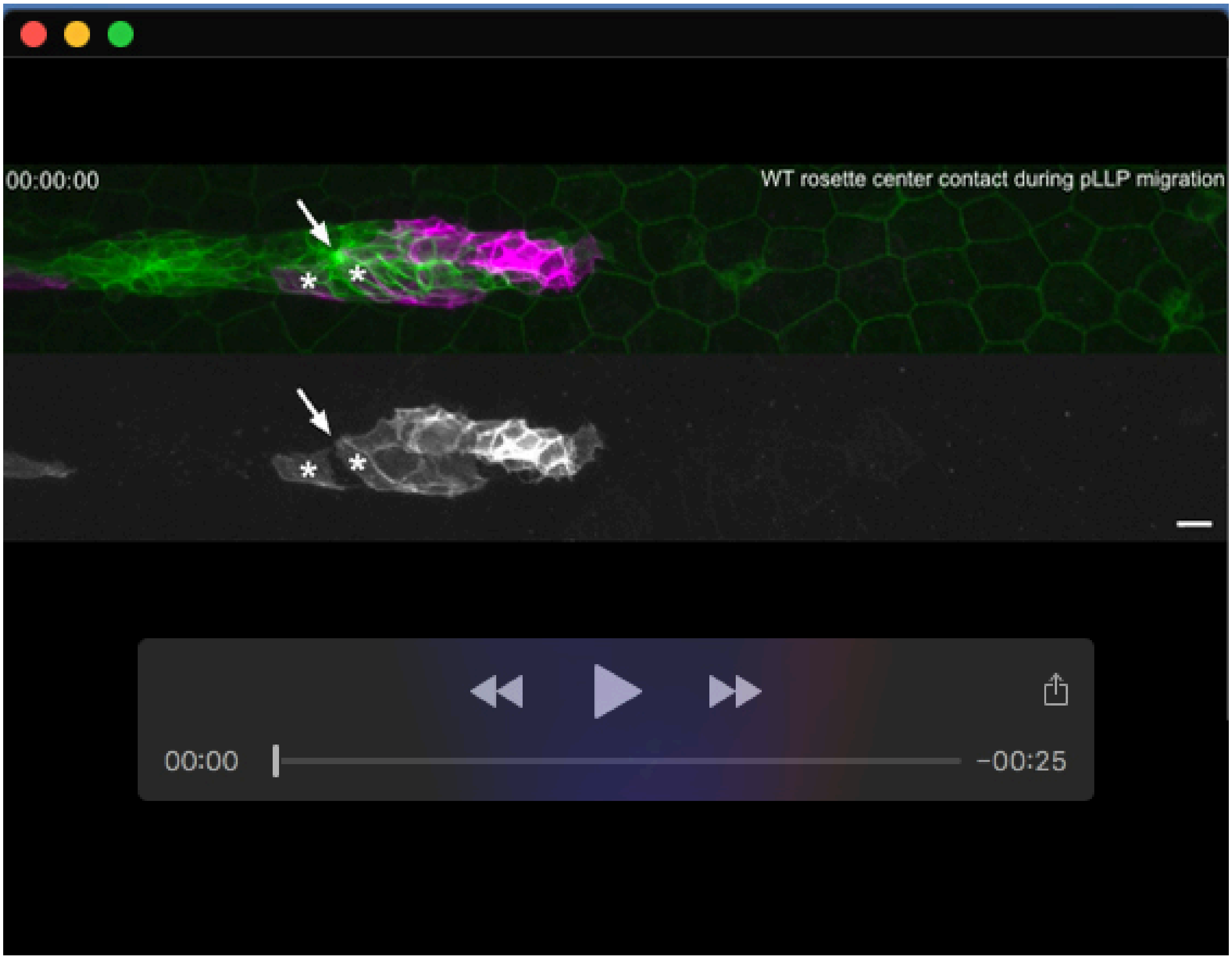

Movie 5. WT cells maintain contact with rosette center during pLLP migration. Time lapse confocal projections of a mosaically labeled WT embryo with WT Tg(*prim:lyn2-mCherry*) positive cells. Embryo was imaged continuously starting at 30 hpf for 1 hours. Scale bar = 10 µm.

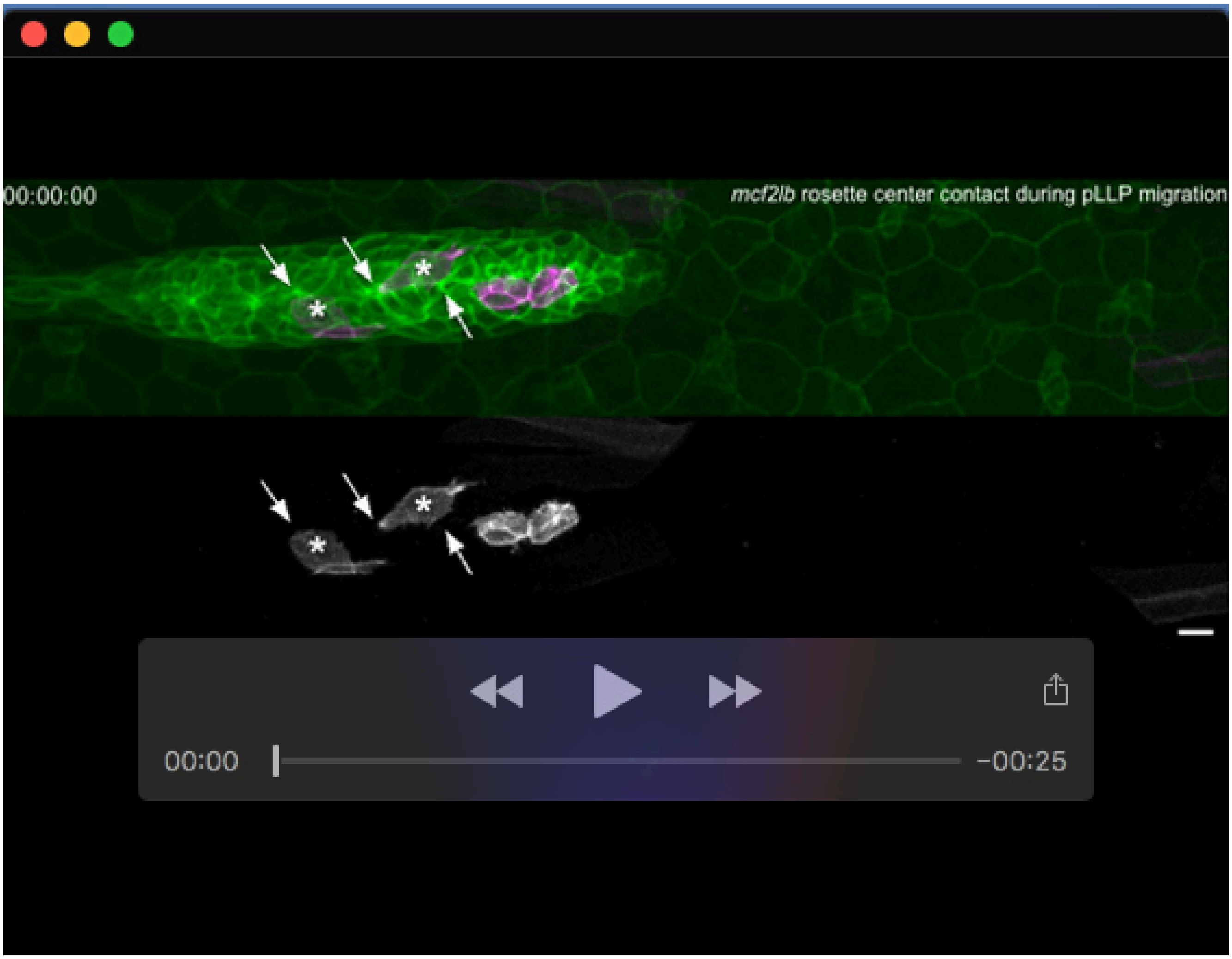

Movie 6. *mcf2lb* mutant cells fail to maintain contact with rosette center during pLLP migration. Time lapse confocal projections of a mosaically labeled *mcf2lb* embryo with the *mcf2lb* Tg(*prim:lyn2-mCherry*) positive cells. Embryo was imaged continuously starting at 30 hpf for 1.5 hours. Scale bar = 10 µm.
