# Supplementary material for: RhoA GEF Mcf2lb regulates rosette integrity during collective cell migration
Source: Development. 2024 Jan 2;151(1):dev201898. doi: 10.1242/dev.201898 (PMC10820872; doi:10.1242/dev.201898)
Supplement: Supplementary file 1 [file develop-151-201898_review_history.pdf]

RhoA GEF Mcf2lb regulates rosette integrity during collective cell migration

Hannah M. Olson, Amanda Maxfield, Nicholas L. Calistri, Laura M. Heiser, Weiyi Qian, Holger Knaut and Alex V. Nechiporuk
DOI: 10.1242/dev.201898

Editor: Thomas Lecuit

Review timeline

Original submission:	20 April 2023
Editorial decision:	12 June 2023
First revision received:	6 October 2023
Editorial decision:	8 November 2023
Second revision received:	19 November 2023
Accepted:	21 November 2023

Original submission

First decision letter

MS ID#: DEVELOP/2023/201898

MS TITLE: RhoA GEF Mcf2lb regulates rosette integrity during collective cell migration

AUTHORS: Hannah M Olson, Amanda Maxfield, Nicholas L Calistri, Laura M Heiser, and Alex V Nechiporuk

I apologize for the delay in coming back to you. I have now received all the referees reports on the above manuscript, and have reached a decision. The referees' comments are appended below, or you can access them online: please go to BenchPress and click on the 'Manuscripts with Decisions' queue in the Author Area.

The overall evaluation is very positive and we would like to publish a revised manuscript in Development, provided that the referees' comments can be satisfactorily addressed. Please attend to all of the reviewers' comments in your revised manuscript and detail them in your point-by-point response. If you do not agree with any of their criticisms or suggestions explain clearly why this is so. If it would be helpful, you are welcome to contact us to discuss your revision in greater detail. Please send us a point-by-point response indicating your plans for addressing the referee's comments, and we will look over this and provide further guidance.

Reviewer 1

Advance summary and potential significance to field

In this manuscript, the authors show that RhoA GEF Mcf2lb regulates the integrity of multicellular rosettes during the collective migration of the posterior lateral line primordium in Zebrafish. It is a

© 2024. Published by The Company of Biologists under the terms of the Creative Commons Attribution License (https://creativecommons.org/licenses/by/4.0/).

significant contribution to the understanding of lateral line migration, and it is very relevant to the field as multicellular rosettes are observed in several developmental contexts.

Comments for the author

The conclusions are supported by the data, but the manuscript could be improved.

Most relevant points:

1. Fig 3: the authors claim that the first neuromast (NM) does not have a significantly different number of cells in WT and mcf2lb mutants, suggesting “that mcf2lb does not play a role in regulating NM size until pLLP migration begins”.
However, the number of embryos used for these quantifications is rather small and there seems to be a tendency for less cells in the mutant. The authors could clarify this by increasing their sample size.

2. The authors show that hair cell differentiation markers are not affected by mcf2lb loss of function. However, the differentiation markers could be independent of rosette formation but differences in cell rearrangements still affect hair cell morphology. Is hair cell morphology different between mutant and WT?

3. Fig 4H,I: are the embryos quantified for foci and points the same? If so, is there a correlation between the number foci and the number of points, e.g. does the number of points increase as the number of foci decreases or vice-versa?

4. Fig 5F: how did the authors perform the statistical analysis for these data?
When data come in proportions, the most suitable statistical tests are typically Chi-square tests or equivalent and not t tests/Mann Whitney test.

5. Fig 5H,I: what were the criteria for using a Mann-Whitney test here? Authors should clarify in the methods the criteria for choosing t tests or Mann-Whitney in different experiments.

6. Fig 5C,D: could the authors measure cell volume? Could a change in this parameter also contribute to defects in apical constriction?

7. Supp Fig 5: the authors say in the text that a microlumen forms in the rostral most rosette, but in Supp Fig 5C,D they label the rosette as NM2. Does NM2 also form a microlumen? Which NM was used for the quantifications? Also, the difference between protoNMs and deposited NM2 should be explained.

8. Fig 6D: the graph does not seem to clearly show what the authors claim in the text, “that the apical membranes of mutant cells are much more variable than WT cells over the course of the imaging period”; also in this graph the apical width is higher in WT than in mutants, contrasting what is shown in Fig 5. This should be clarified.

9. Fig 6: it seems that the data on apical membrane variability may be biased because the apical width of the mutant should be higher than the WT. If so, this analysis does not seem to add much to manuscript and, taking into account that Fig 5 is sufficiently convincing regarding the differences in apical constriction between WT and mutant, Fig 6 could even be removed from the final version of the manuscript or included as a supplementary figure. I understand that this experiment must have been very laborious, but to include it as a major figure the authors need to justify it more clearly.

10. Fig 8C,D,G,H: is not competently clear how these quantifications were made.
Are the values normalized to fluorescence intensity and number of pixels in the rest of the cell/other cells?

© 2024. Published by The Company of Biologists under the terms of the Creative Commons Attribution License (https://creativecommons.org/licenses/by/4.0/).

11. Discussion: the authors claim that they have evidence that Mc2lb regulates RhoA, which subsequently activates Rock and Myo2. However, the evidence presented is indirect. Also, it cannot be excluded that Mc2lb does act via Cdc42 (or Rac).
In fact, Cdc42 is known to regulate apical polarity in several contexts, and from the authors' data it seems highly expressed in follower cells (Supp Fig 1A).
Therefore, the authors could look into more direct methods of assessing Rho GTPase activation, e.g. Rho/Cdc42 activity sensors, or be more conservative in their claims.

12. Discussion: how do the authors envision that apical constriction leads to increased NM deposition? Could it be that cell adhesion between follower cells and the surrounding cells is misregulated, making it easier for followers to detach from pLLP?

Minor points:

1. Line numbers are not completely visible in the document, making it difficult to mark comments.

2. Fig 1: authors describe that wnt components are upregulated in cluster 1 (leader cells), but they also seem to be upregulated in proliferating cells (cluster 0). Is that correct? Can you clarify this? Also, can you explain where in pLLP are the proliferating cells? Are they part of both leading and follower cells?

3. Fig 2 and Supp Figs resolution is very low, especially the graphs. This should be improved for better visualization in the final version.

4. Supp Fig 5 legend spelling mistakes: "integrity", "magnification", "intensity"

5. In the description of the analyses of apical polarity (Figs 7,8), the authors refer to "90 degree digital rotation", which is ambiguous; a more common and specific description should be used, such as cross-section or XZ/YZ slice. Along the same lines, the authors should mention in the legend which images they show (Z slice, Z projection, etc).

Reviewer 2

Advance summary and potential significance to field

Olson and colleagues present evidence for a role of Mcf2lb in epithelial rosette homeostasis. The problem is interesting. The used approach is correct and the quality of the data is excellent. My comments are for minor improvements.
One thing that is surprising is that many of the references are very old. I am supportive of citing original descriptions that remain relevant regardless of their age. However, the authors use many dated publications that have been superseded by various other papers. For instance, Montgomery 2000, Metcalfe 1985 Dambly-Chaudiere 2004, Chen and Macara 2005 should be, in my opinion, replaced by newer work.

Pages are not numbered, which made the revision cumbersome.

It seems that the authors cannot decide if Mcf2lb is needed for rosettogenesis or to maintain rosette integrity. On this, it would be helpful if the authors tried to produce a scheme of the model that they propose. The panels in Figure 8 may be size-reduced to accommodate a scheme.

The authors indicate smaller neuromasts in Mcf2lb mutant fish. However, what they obtain is neuromasts with fewer cells. This should be clarified to disambiguate between neuromasts with fewer cells versus neuromasts of smaller volume or occupied area.

The authors define significance using wildly variable P values in their t-tests.

© 2024. Published by The Company of Biologists under the terms of the Creative Commons Attribution License (https://creativecommons.org/licenses/by/4.0/).

Why is it the case?

What do the authors mean by "cell polarity is largely normal in Mcf2lb mutant pLLP? The way this is written is meaningless because "largely normal" is open to subjective interpretation. If they cannot provide any quantitative measure, they should eliminate the statement.
Same goes for "slightly impaired" organization of ZO-1.
Moreover, in the same page the authors state that "...mcf2lb mutant pLLP are normally polarized".

I do not understand the reason behind discussing the pulling-pushing issue during PLL migration, when the work focuses on rosette homeostasis.

Comments for the author

kono-cilia should be kinocilia.

Reviewer 3

Advance summary and potential significance to field

The paper by Olson et al describes single cell RNA analysis of cells from the zebrafish pLLP, which clusters cells into three populations: “leaders”, “followers” and “proliferating” cells. The study goes on to identify genes primarily expressed in genes in the “follower” clusters that regulate actin polymerization and apical constriction to pursue the studies specific interest in understanding the mechanisms that regulate epithelial rosette formation in the primordium. The study examines expression of mcf2lb, twf2b, arhgef4 and fhdc1 confirming predictions of the single cell analysis and then the study goes on to focus on analysis of mcf2lb function in promoting apical constriction for most of the study. They show that in the absence of mcf2lb function, while apical basal polarization is not affected, apical constriction is severely compromised. This affects effective morphogenesis of stable epithelial rosettes within the migrating primordium and results in an aberrant pattern of neuromast deposition. The authors develop a series of effective measures to quantify and compare many features of apical constriction and epithelial rosette morphogenesis in wild-type and mutant primordia. The authors show that compromised apical constrictions is associated with aberrant localization of ZO-1 and Par3. Furthermore, these changes are associated with compromised RhoA as evidenced by aberrant distribution of Rock-2a and importantly reduction/loss of pMRLC. The study overall is well laid out, well documented and the conclusions for the most part, are consistent with their observations.

Comments for the author

I had a few questions and thought some clarifications would help improve the study.

1.Please provide the reference that identifies hmx2 and hmx3a as known lateral line makers, it will be useful to readers (I was not aware of it and looked it up!).
2. Was there a zygotic phenotype or was the phenotype only seen in maternal zygotic mutants?
3. What is the pattern of atoh1a and DeltaA in the primordium. How does their number and location correspond to the pattern of constriction and number of deposited cells. What is the pattern of pea3? Is atoh1a contributing to the pattern of deposition and neuromast formation or does the link become dissociated? The current thinking is that specification of the central sensory hair cell progenitor and morphogenesis of surrounding cells to form epithelial rosettes is coordinated and linked. It would be useful to know what happens to this relationship-4. It’s not clear that the microlumen forms in the mutant at all- the fluorescence shown could be of the cell. How was the presumptive outline of the microlumen defined?
5. There appears to be some more basal ZO-1 in Fig.7F’ and H’. Could you quantify if there any difference in basal ZO-1 in wild-type and mutant- that might be a more sensitive way to quantify the difference that seems apparent in the images referenced above.

© 2024. Published by The Company of Biologists under the terms of the Creative Commons Attribution License (https://creativecommons.org/licenses/by/4.0/).

6. Rosette integrity does not seem to affect deposition per se just the deposition of stable rosettes whose formation has already been established to be compromised. In fact, previous studies that suggest the importance of lumen formation, FGF signaling, and stable neuromast morphogenesis for "deposition" also seem to confuse mechanisms that determine deposition of stable neuromasts with mechanisms that determine more generally mechanisms that determine deposition, ie when primordium cells lose their capacity to migrate collectively and are deposited. If "deposition" was indeed affected more generally, would it not be expected to alter the length of the primordium; lengthened if deposition is delayed and shortened if accelerated. On the other hand, to say neuromast deposition is affected in situations where epithelial rosette morphogenesis is compromised seems redundant- if they are not formed properly, they could not be expected to be deposited "normally"?

First revision

<u>Author response to reviewers' comments</u>

<u>Response to Reviewer 1</u>

Major Points

Point 1: Fig. 3 – The authors claim that the first neuromast (NM) does not have a significantly different number of cells in WT and mcf2lb mutants, suggesting "that mcf2lb does not play a role in regulating NM size until pLLP migration begins". However, the number of embryos used for these quantifications is rather small and there seems to be a tendency for less cells in the mutant. The authors could clarify this by increasing their sample size.

> Response: As the reviewer suggested, we increased the sample size to n = 19 for wildtypes and n = 25 for *mcf2lb* mutants. After increasing the sample size, we confirmed that the *mcf2lb* NM1 cell number is not significantly different from that of WT. The graph in Fig. 3, the text on Page 15, and the figure legend on Page 25 has been updated to reflect new data.

Point 2: The authors show that hair cell differentiation markers are not affected by mcf2lb loss of function. However, the differentiation markers could be independent of rosette formation but differences in cell rearrangements still affect hair cell morphology. Is hair cell morphology different between mutant and WT?

> Response: To address this question, we measured hair cell volume, apical width, and basal width in deposited L5 NM in WT and *mcf2lb* mutant embryos. We found no significant difference in cell volume and apical width of hair cells in deposited NMs when comparing WT and *mcf2lb* mutants. However, we did find a significant decrease in the basal width of hair cells between WT and *mcf2lb* mutants. Despite no statistical differences, the average apical width in mutants was higher (4.4 μm in the mutant vs. 3.2 μm in WT) and had a much higher variability compare to wildtypes. These data indicate that *mcf2lb* mutant hair cells differentiate properly and maintain proper cell volume but do have altered morphology when compared to wildtype hair cells. We added these results to Supplemental Fig. 5 and described on Page 17.

Point 3: Fig 4H,I – Are the embryos quantified for foci and points the same? If so, is there a correlation between the number foci and the number of points, e.g. does the number of points increase as the number of foci decreases or vice-versa?

> Response: Yes, the embryos quantified for foci and points are the same. Gatherings of membranes were determined to be a focus if the width and the heigh of the focus was

© 2024. Published by The Company of Biologists under the terms of the Creative Commons Attribution License (https://creativecommons.org/licenses/by/4.0/).

within one standard deviation above or below the average of all WT foci. If the gathering of membrane did not meet these criteria, it was determined to be a point. And the reviewer is correct: there is a correlation between the number of foci and number of points. As the number of points increase the number of foci decrease. We added an additional graph for clarification and it is now Panel J in Figure 4. We also updated the Figure legend on page 26 as well as the text on Page 16.

Point 4: Fig 5F – How did the authors perform the statistical analysis for these data? When data come in proportions, the most suitable statistical tests are typically Chi-square tests or equivalent and not t tests/Mann Whitney test.

Response: We agree with the Reviewer's comment and re-analyzed the data using the appropriate statistical test, the chi-square test for proportions (categorical) data. The corresponding results still remained statistically significant. We modified the Methods to reflect these changes (Page 12) as well as the figure legends (Page 26).

Point 5: Fig 5H,I – What were the criteria for using a Mann-Whitney test here? Authors should clarify in the methods the criteria for choosing t tests or Mann-Whitney in different experiments.

Response: To address this comment, we added details to the Statistics section within the Methods regarding how particular statistical tests were used (Page 12). Experimental data was analyzed for normality using a Shapiro-Wilk test and Q-Q plots (e.g. parametric versus nonparametric). If data were determined to be parametric, we applied unpaired t-tests. If data were determined to be nonparametric, we used a Mann Whitney U test (nonparametric alternative to unpaired t-tests) or Kruskal – Wallis (nonparametric alternative to ANOVA). For categorical variables described as proportions, we used a Chi-square test.

Point 6: Fig 5C,D – Could the authors measure cell volume? Could a change in this parameter also contribute to defects in apical constriction?

Response: To address this point, we measured volume of cells in the most trailing rosette within wild-type and *mcf2lb* mutant pLLPs. Following analysis, we found there was no significant difference in cell volume between wildtypes and *mcf2lb* mutants (Fig. 5H, page 18). Therefore, while cell volume was maintained within the *mcf2lb* mutants the morphology, specifically the cells ability to apically constrict, was diminished.

Point 7: Supp Fig 5 – The authors say in the text that a microlumen forms in the rostral most rosette, but in Supp Fig 5C,D they label the rosette as NM2. Does NM2 also form a microlumen? Which NM was used for the quantifications? Also, the difference between protoNMs and deposited NM2 should be explained.

Response: We apologize for the confusion. Supp Fig. 5 is now Supp Fig 6. In Supp Fig. 6, panels A and B are images from the rostral most rosette in the migrating pLLP. Panels C and D are images of the most recently deposited NMs. To clarify this, we added additional labels to these panels. In WT, the microlumen forms in the trailing most rosette and is still present in the most recently deposited rosette. Both the trailing rosette and NM2 were used for quantification. We clarified the language and removed the term protoNM as that term had not previously been used in the manuscript.

Point 8: Fig 6D – The graph does not seem to clearly show what the authors claim in the text, "that the apical membranes of mutant cells are much more variable than WT cells over the course of the imaging period"; also in this graph the apical width is higher in WT than in mutants, contrasting what is shown in Fig 5. This should be clarified.

Response: We apologize for the mix up: the WT and *mcf2lb* mutant labels were

© 2024. Published by The Company of Biologists under the terms of the Creative Commons Attribution License (https://creativecommons.org/licenses/by/4.0/).

accidentally switched on Fig. 6D in the initial manuscript submission. The labels have been corrected and the figure has been updated. The figure now accurately portrays what was discussed in the text, i.e., the apical region of mutant cells is more variable than that of wild-type cells.

Point 9: It seems that the data on apical membrane variability may be biased, because the apical width of the mutant should be higher than the WT. If so, this analysis does not seem to add much to manuscript and, taking into account that Fig 5 is sufficiently convincing regarding the differences in apical constriction between WT and mutant, Fig 6 could even be removed from the final version of the manuscript or included as a supplementary figure. I understand that this experiment must have been very laborious, but to include it as a major figure the authors need to justify it more clearly.

Response: As we noted above, we made an error annotating the figure. The mutant apical variability is much higher than that of the wildtype. Thus, Fig. 6 provides important data regarding the dynamic nature of the apical constriction process in a live embryo, whereas the data and experiments shown in Fig. 5 provide only snapshots of this process. As such, we believe it is important to illustrate these data in the manuscript.

Point 10: Fig 8C,D,G,H – It's not competently clear how these quantifications were made. Are the values normalized to fluorescence intensity and number of pixels in the rest of the cell/other cells?

Response: Figure 8 is now Figure 9. To address this point, we added additional clarification to the methods section (Page 11, 12). For Fig. 9 panel C and G, fluorescence intensity signal for either Rock-2a or pMRLC, respectively, was measured throughout the entire pLLP. That value was then normalized to the size of the pLLP by dividing that value by the number of cells within the pLLP from which the measurement came from. For Fig. 9 panel D and H, fluorescence intensity signal for either Rock-2a or pMRLC, respectively was measured at rosette centers or gatherings of membranes using a consistently sized ROI. The ROI was determined by using the average width and length of a focus and one-third of the average depth of the pLLP (Rock-2a dimensions = 2.6μm X 1.65μm X 5.6μm; pMRLC dimensions = 3.78μm X 2.12μm X 5.2μm). These values are presented as individual measurements and are not normalized.

Point 11: Discussion – The authors claim that they have evidence that Mc2lb regulates RhoA, which subsequently activates Rock and Myo2. However, the evidence presented is indirect. Also, it cannot be excluded that Mc2lb does act via Cdc42 (or Rac). In fact, Cdc42 is known to regulate apical polarity in several contexts, and from the authors' data it seems highly expressed in follower cells (Supp Fig 1A). Therefore, the authors could look into more direct methods of assessing Rho GTPase activation, e.g. Rho/Cdc42 activity sensors, or be more conservative in their claims.

Response: To address this point, we utilized an RhoA sensor line, TgBAC (*cxcr4b:AHPH-GFP)* to examine RhoA activity at rosette centers (Fig. 8; Page 20, 21). In this transgenic line, the RhoA binding domain of the protein Anillin is fused to GFP. This domain binds active RhoA, which leads to the localized accumulation of fluorescence. In *mcf2lb* mutant pLLP, we found a significant reduction (or in many cases a complete absence) of the sensor signal at rosette centers. This provides direct evidence that a lack of active RhoA at the rosette centers results in impaired apical constriction.

Also, The Reviewer is correct in commenting that other Rho GTPases could also be playing role in rosette formation. In support of this, pLLP cells are still partially constricted even in the absence of the RhoA activation. Indeed, Cdc42 is reported to activate apical constriction in a number of contexts. We added additional text to the discussion to address this possibility (Page 22).

Point 12: Discussion – How do the authors envision that apical constriction leads to increased NM

© 2024. Published by The Company of Biologists under the terms of the Creative Commons Attribution License (https://creativecommons.org/licenses/by/4.0/).

deposition? Could it be that cell adhesion between follower cells and the surrounding cells is misregulated, making it easier for followers to detach from pLLP?

Response: We appreciate this Reviewers comment. We hypothesize that impaired apical constriction in the pLLP leads to a lack of integrity of rosette structures leading to the formation of additional small rosettes. However, the process of the deposition pers se is not probably affected (please see our comments to Reviewer 3). As a result, the pLLP deposits multiple, often smaller rosettes together instead of a normal size, singular rosette. We cannot exclude a possibility that cellular adhesion within the trailing region of the pLLP is disrupted. Although, we would expect a more obvious cell dispersal observed in cadherin mutants or other mutants causing the disruption of cell adhesion.

Minor Points

Point 1: Line numbers are not completely visible in the document, making it difficult to mark comments.

Response: Line numbers were adjusted to be more visible.

Point 2: Fig 1 – Authors describe that wnt components are upregulated in cluster 1 (leader cells), but they also seem to be upregulated in proliferating cells (cluster 0). Is that correct? Can you clarify this? Also, can you explain where in pLLP are the proliferating cells? Are they part of both leading and follower cells?

Response: That is correct. Previous studies show that proliferating cells are found in both the leading and trailing regions of the pLLP (Dalle Nogare et al., 2017). Thus, it is not surprising that the proliferating cell cluster shares some similarity in gene expression with the other two clusters. We added a few sentences to clarify this point (Page 13).

Point 3: Fig 2 and Supp Figs resolution is very low, especially the graphs. This should be improved for better visualization in the final version.

Response: In the revised manuscript, all main figures were saved at a much higher resolution (300-600 dpi) and uploaded individually. Supplemental figures were also saved at higher resolution and included in the revised supplemental materials file.

Point 4: Supp Fig 5 – Legend spelling mistakes: “integrity”, “magnification”, “intensity”

Response: We corrected this mistake. Supplemental Fig. 5 is now Supplemental Fig. 6.

Point 5: In the description of the analyses of apical polarity (Figs 7,8), the authors refer to “90 degree digital rotation”, which is ambiguous; a more common and specific description should be used, such as cross-section or XZ/YZ slice. Along the same lines, the authors should mention in the legend which images they show (Z slice, Z projection, etc).

Response: To address this point, we added dotted lines along the X-axis of the pLLP to indicate where the rotation occurred. Additionally, we specified in the figure legend (Page 27, 28) that these images were Z projections.

Response to Reviewer 2

Minor Points

Point 1: One thing that is surprising is that many of the references are very old. I am supportive of citing original descriptions that remain relevant regardless of their age. However, the authors use many dated publications that have been superseded by various other papers. For instance, Montgomery 2000, Metcalfe 1985, Dambly-Chaudiere 2004, Chen and Macara 2005 should be, in my

© 2024. Published by The Company of Biologists under the terms of the Creative Commons Attribution License (https://creativecommons.org/licenses/by/4.0/).

opinion, replaced by newer work.

Response: To address this point, the above references were replaced with more recent ones in the revised manuscript.

Point 2: Pages are not numbered, which made the revision cumbersome.

Response: We have added page numbers to the revised manuscript.

Point 3: It seems that the authors cannot decide if Mcf2lb is needed for rosettogenesis, or to maintain rosette integrity. On this, it would be helpful if the authors tried to produce a scheme of the model that they propose. The panels in Figure 8 may be size-reduced to accommodate a scheme.

Response: Indeed, we were careful in our conclusions as to whether *mcf2lb* was required for rosettogenesis and/or to maintain rosette integrity. While our data, specifically the live measurements of apical constriction, strongly argue for the Mcf2lb's role in maintaining rosette integrity, we cannot exclude the possibility that Mcf2lb is also required for the initial rosette formation. Therefore, we chose to be careful in defining *mcf2lb*'s role in these two processes.

While we appreciate the Reviewers suggestion about the model, this manuscript has already exceeded the allowed length for a typical manuscript in Development. Additionally, the process of apical constriction during pLLP migration is difficult to depict in 2D. For these reasons, we have opted to not add a scheme.

Point 4: The authors indicate smaller neuromasts in Mcf2lb mutant fish. However, what they obtain is neuromasts with fewer cells. This should be clarified to disambiguate between neuromasts with fewer cells versus neuromasts of smaller volume or occupied area.

Response: To address this point, we changed the language describing the difference observed in NM size when comparing WT and *mcf2lb* mutant embryos (Page 15).

Point 5: The authors define significance using wildly variable P values in their t-tests. Why is it the case?

Response: We appreciate the Reviewers comment and have edited the manuscript for more uniform p values. Additionally, we provided an additional explanation in the statistics section within the methods about statistical tests and p values (Page 12).

Point 6: What do the authors mean by "cell polarity is largely normal in Mcf2lb mutant pLLP? The way this is written is meaningless because "largely normal" is open to subjective interpretation. If they cannot provide any quantitative measure, they should eliminate the statement. Same goes for"slightly impaired" organization of ZO-1. Moreover, in the same page the authors state that "...mcf2lb mutant pLLP are normally polarized".

Response: We appreciate the Reviewers comment and have adjusted the language within the text to be less ambiguous. The terms “largely normal”, “slightly impaired”, and “normally polarized” have been replaced with clearer language (Page 19, 20).

Point 7: I do not understand the reason behind discussing the pulling-pushing issue during PLL migration, when the work focuses on rosette homeostasis.

Response: This brief discussion is related to the observation, based on our scRNA-seq data, showing that most factors regulating actin dynamics are expressed at higher levels in the trailing region. As this is part of our study, we commented that this observation is

© 2024. Published by The Company of Biologists under the terms of the Creative Commons Attribution License (https://creativecommons.org/licenses/by/4.0/).

consistent with a previously proposed model (Yamaguchi et al., 2022). We removed this paragraph from the discussion and included a brief conclusion in the results text on Page 14.

Point 8: kono-cilia should be kinocilia.

Response: We have replaced kono-cilia with kinocilia.

<u>Response to Reviewer 3</u>

Minor Points:

Point 1: Please provide the reference that identifies hmx2 and hmx3a as known lateral line makers, it will be useful to readers (I was not aware of it and looked it up!).

Response: We have added the reference (Feng and Xu, 2010) to the revised manuscript (Page 13).

Point 2: Was there a zygotic phenotype or was the phenotype only seen in maternal zygotic mutants?

Response: Yes, there is a zygotic phenotype and we added the quantification of that phenotype to Supplemental Figure 3. However, the cellular phenotype observed within the migrating pLLP is more pronounced in maternal zygotic homozygous mutants.

Point 3: What is the pattern of atoh1a and DeltaA in the primordium? How does their number and location correspond to the pattern of constriction and number of deposited cells? What is the pattern of pea3? Is atoh1a contributing to the pattern of deposition and neuromast formation or does the link become dissociated? The current thinking is that specification of the central sensory hair cell progenitor and morphogenesis of surrounding cells to form epithelial rosettes is coordinated and linked. It would be useful to know what happens to this relationship.

Response: To address this point, we performed in situ hybridization in wildtype and *mcf2lb* mutant embryos at 30 hpf using probes against *atoh1a*, *deltaA*, and *etv4* (*pea3*). (Supplemental Fig. 4). We observed a significant increase in *atoh1a* positive foci within the *mcf2lb* pLLP compared to WT, which is likely due to the presence of points (i.e. additional small rosettes) within *mcf2lb* mutant pLLPs.

Interestingly, there is no significant difference in the number of deltaA positive foci within the migrating pLLP. Although, we noted that 5 out 23 mutant pLLPs had more *deltaA* foci (>3) as well as more *deltaA*+ cells per foci. However, these results were not statistically significant (Supplemental Fig. 4D).

Finally, to analyze the *etv4* expression, we quantified the ratio of the *etv4* domain to the length of the pLLP in wildtypes and mutant. We found no significant difference between WT and *mcf2lb* mutant *pea3* (etv4) domains (Supplemental Fig. 4A,B, G).

These results indicate that while the Fgf signaling domain is not altered, there is an increase of the hair cell specification marker, *atoh1a*. Thus, the link between the central foci and rosette morphogenesis persists even within smaller mutant rosettes. This is also consistent with our observation that almost all deposited NMs contain both *atoh1a*- and *deltaA*-positive cells. This explanation is in the text on Page 16 & 17.

Point 4: It's not clear that the microlumen forms in the mutant at all- the fluorescence shown could be of the cell. How was the presumptive outline of the microlumen defined?

© 2024. Published by The Company of Biologists under the terms of the Creative Commons Attribution License (https://creativecommons.org/licenses/by/4.0/).

Response: Indeed, our secGFP data strongly argue that microlumen does not form in the mutant. However, we cannot be absolutely sure without performing additional experiments: for example, visualizing microlumen integrity via transmission electron microscopy. We have added this point in the revised manuscript (Page 23).

As we cannot determine the microlumen outline solely based on memGFP expression in pLLP cells, we used a consistently sized circular ROI (1 μm in diameter) placed in the center of the foci (or points) to measure fluorescence intensity (Supplemental Fig. 6). This is detailed in the methods section on Page 11.

Point 5: There appears to be some more basal ZO-1 in Fig.7F' and H'. Could you quantify if there any difference in basal ZO-1 in wild-type and mutant- that might be a more sensitive way to quantify the difference that seems apparent in the images referenced above.

Response: To address this comment, we quantified the percentage of both apical and basal signals. The results were consistent to our prior analysis: ZO-1 is still apically, but not basally, localized in the mutant pLLP; although, there is a significant accumulation of the basal ZO-1 in mutant NMs. We replaced the graphs in Fig. 7K, L to better reflect the distribution of apical and basal ZO-1 signal.

Point 6: Rosette integrity does not seem to affect deposition per se just the deposition of stable rosettes whose formation has already been established to be compromised. In fact, previous studies that suggest the importance of lumen formation, FGF signaling, and stable neuromast morphogenesis for “deposition” also seem to confuse mechanisms that determine deposition of stable neuromasts with mechanisms that determine more generally deposition, ie when primordium cells lose their capacity to migrate collectively and are deposited. If “deposition” was indeed affected more generally, would it not be expected to alter the length of the primordium; lengthened if deposition is delayed and shortened if accelerated. On the other hand, to say neuromast deposition is affected in situations where epithelial rosette morphogenesis is compromised seems redundant- if they are not formed properly, they could not be expected to be deposited “normally”?

Response: We do agree with this comment, i.e. rosette deposition per se does not seem to be affected, as both normally and abnormally sized clusters are deposited, albeit at abnormal intervals. As the reviewer pointed out, this is likely due to the rosette morphogenesis defect, which in this case is primary. We clarified this point in the revised manuscript (page 23).

In summary, we believe we adequately addressed all reviewers' comments in the revised version of our manuscript. Please do not hesitate to let us know if we can provide any more information in support of our work for publication in *Development*.

<u>Second decision letter</u>

MS ID#: DEVELOP/2023/201898

MS TITLE: RhoA GEF Mcf2lb regulates rosette integrity during collective cell migration

AUTHORS: Hannah M Olson, Amanda Maxfield, Nicholas L Calistri, Laura M Heiser, Weiyi Qian, Holger Knaut, and Alex V Nechiporuk

© 2024. Published by The Company of Biologists under the terms of the Creative Commons Attribution License (https://creativecommons.org/licenses/by/4.0/).

I have now received all the referees reports on the above manuscript, and have reached a decision. The referees' comments are appended below, or you can access them online: please go to BenchPressand click on the 'Manuscripts with Decisions' queue in the Author Area.

The overall evaluation is very positive and we would like to publish your manuscript in Development, provided that you address the comments from Reviewer 3 by editing the text as suggested for clarity.

Reviewer 1

Advance summary and potential significance to field

In this manuscript, the authors show that RhoA GEF Mcf2lb regulates the integrity of multicellular rosettes during the collective migration of the posterior lateral line primordium in Zebrafish. It is a significant contribution to the understanding of lateral line migration, and it is very relevant to the field as multicellular rosettes are observed in several developmental contexts.

Comments for the author

Reviewers comments have been addressed.

Reviewer 2

Advance summary and potential significance to field

The paper assigns a role of the RhoA GEF Mcf2lb in rosette morphogenesis.

Comments for the author

The authors state in their rebuttal letter that the want to be careful about the whether Mcf2lb is important for rosette formation and/or maintenance. However, in the abstract they keep claiming that the RhoA GEF Mcf2lb is needed for rosette morphogenesis, which implied formation/development. Can they clarify or take a stand?

No additional comment, other than to maintain my encouragement for the authors to draft a scheme to explain their findings.

Reviewer 3

Advance summary and potential significance to field

The strength of this paper is in the collection and analysis of single cell data which helped identify 3 populations within the migrating primordium with distinct transcription profiles and the analysis of genes that are especially important for apical constriction. The quantification and demonstration, now with the added RhoA sensor data, of the link between Mcf2lb function, RhoA activation and apical constriction, is excellent.

Comments for the author

The authors have addressed all the issues I had raised and examined atoh1a, deltaA and etv4 as requested to better understand the relationship between the aberrant pattern of rosette formation and the pattern of Fgf signaling that initiates rosette formation. A concern, however, is that the new observations are hard to understand and there are many seemingly paradoxical and hard to reconcile observations which raise the possibility that the effects mcf2lb loss on rosette formation and neuromast deposition are more complicated than suggested by the author’s description of their results and associated discussion.

© 2024. Published by The Company of Biologists under the terms of the Creative Commons Attribution License (https://creativecommons.org/licenses/by/4.0/).

1. My expectation was that the mcf2lb mutation, which affects the mechanics of apical constriction, will affect morphogenesis of epithelial rosettes in the migrating primordium but not the underlying pattern of Fgf signaling that initiates their formation. The described observations, however, suggest unexpected effects on the underlying signaling systems. The authors suggest that Fgf signaling, as assayed by etv4, is unchanged. However, the in-situ staining provided in supplementary data is light and the contrast is not adequate to be sure of this. Second, the number of atoh1a positive cells is increased. If Fgf signaling is unchanged there is no obvious reason for this to happen as Fgf signaling initiates atoh1a expression, while Notch signaling keeps it restricted to a single cell. Is altered rosette formation somehow affecting the underlying signaling in a manner that increases the number of sensory hair cell progenitors?
The authors also suggest that while mcf2lb mutants have no change in etv4 there is an increase in the number of deltaA expressing cells. This too is confusing as both DeltaA and DeltaD mediate lateral inhibition to keep atoh1a expression restricted to a central cell sensory hair cell progenitor in a nascent neuromast.
The literature suggests that knock down of atoh1 homologues results in loss of deltaD expression, not deltaA. Inhibition of Fgf signaling does, however eliminate deltaA expression suggesting its expression is ultimately dependent on Fgf signaling, not atoh1a. Hence the increase in the number of deltaA expressing cells raises the possibility that there may after all be a change in the pattern of Fgf signaling or other there are other reasons for the change in number of deltaA expressing cells.
2. Fgf signaling normally helps coordinate morphogenesis of epithelial rosettes with specification a central sensory hair cell progenitor in periodically forming neuromasts within the migrating primordium. The examination of rosette formation with the quantification of foci within the migrating primordium and their correlation with the pattern of Atoh1a, DeltaA or etv4 helps correlate the periodic pattern of Fgf signaling with periodic morphogenesis of rosettes and deposition of neuromasts. However, it is not clear what the morphogenesis of poorly formed rosettes within the migrating primordium has to do with the ultimate pattern of deposited neuromasts in mcf2lb mutants. Both sets of videos suggest that primordium cells seem to lose their organization as rosettes while depositing, they deposit essentially unclustered, and they seem to re-aggregate post-deposition to re-form rosettes of variable size, though this is hard to see in the higher mag videos that were designed to focus on morphogenesis within the primordium. The authors should address this issue and determine if there indeed a disconnect between the cells that initially aggregate to form poorly formed rosettes in the primordium and how they ultimately reorganize following deposition? If there is a disconnect, what accounts for the patten of aggregation of deposited cells?
The strength of this paper is in the collection and analysis of single cell data which helped identify 3 populations within the migrating primordium with distinct transcription profiles and the analysis of genes that are especially important for apical constriction. The quantification and demonstration, now with the added RhoA sensor data, of the link between Mcf2lb function, RhoA activation and apical constriction, is excellent. Given that the primary focus of the paper is demonstrating the link between mcf2lb function, RhoA and apical constriction, it is not clear that the authors need to account for all the issues raised above related to changes in the number of atoh1a and deltaA expressing cells and the potential disconnect between rosette morphogenesis within the primordium and the ultimate pattern of deposited neuromasts. Nevertheless, these observations and the issues they raise need to be discussed, especially the potential disconnect between rosette morphogenesis within the primordium and ultimate pattern and morphology of deposited neuromasts, to better appreciate the complexity of changes resulting from loss of mcf2lb function. Of course, explanations for some of these observations would significantly improve the impact of the paper.

Second revision

Author response to reviewers' comments

© 2024. Published by The Company of Biologists under the terms of the Creative Commons Attribution License (https://creativecommons.org/licenses/by/4.0/).

Response to Reviewer 2

The authors state in their rebuttal letter that they want to be careful about whether Mcf2lb is important for rosette formation and/or maintenance. However, in the abstract they keep claiming that the RhoA GEF Mcf2lb is needed for rosette morphogenesis, which implied formation/development. Can they clarify or take a stand?

- As the reviewer requested, we "took a stand" and rephrased the last sentenced in the abstract. It now states: "Thus, Mcf2lb-dependent RhoA activation maintains integrity of epithelial rosettes. "

Response to Reviewer 3

1) My expectation was that the mcf2lb mutation, which affects the mechanics of apical constriction, will affect morphogenesis of epithelial rosettes in the migrating primordium but not the underlying pattern of Fgf signaling that initiates their formation. The described observations, however, suggest unexpected effects on the underlying signaling systems. The authors suggest that Fgf signaling, as assayed by etv4, is unchanged. However, the in-situ staining, provided in supplementary data is light and the contrast is not adequate to be sure of this. Second, the number of atoh1a positive cells is increased. If Fgf signaling is unchanged there is no obvious reason for this to happen as Fgf signaling initiates atoh1a expression, while Notch signaling keeps it restricted to a single cell. Is altered rosette formation somehow affecting the underlying signaling in a manner that increases the number of sensory hair cell progenitors? The authors also suggest that while mcf2lb mutants have no change in etv4 there is an increase in the number of deltaA expressing cells. This too is confusing as both DeltaA and DeltaD mediate lateral inhibition to keep atoh1a expression restricted to a central cell sensory hair cell progenitor in a nascent neuromast. The literature suggests that knock down of atoh1 homologues results in loss of deltaD expression, not deltaA. Inhibition of Fgf signaling does, however, eliminate deltaA expression suggesting its expression is ultimately dependent on Fgf signaling, not atoh1a. Hence the increase in the number of deltaA expressing cells raises the possibility that there may after all be a change in the pattern of Fgf signaling or other there are other reasons for the change in number of deltaA expressing cells.

- We thank the reviewer for their thoughtful comments. We think there are two possible explanations for ectopic *atoh1a* and *deltaA* foci. One is an increase in the Fgf signaling due to improperly formed microlumen in the trailing pLLP region, as evidenced by our secreted-GFP experiments. We also recognize that the conventional *etv4* in situ may not be sufficient in detecting subtle differences in the expression pattern. Thus, further experiments using fluorescent in situ hybridization and/or Fgf activity reporter lines are necessary to carefully evaluate the Fgf activity in the *mcf2lb* mutant pLLP. On the other hand, Mcf2lb may have another role in the pLLP besides regulating RhoA activity, especially since *mcf2lb* is widely expressed in the trailing region pLLP. We added sentences to the discussion to explain this in more detail Page 23, Lines 697 -704.

2) Fgf signaling normally helps coordinate morphogenesis of epithelial rosettes with specification a central sensory hair cell progenitor in periodically forming neuromasts within the migrating primordium. The examination of rosette formation with the quantification of foci within the migrating primordium and their correlation with the pattern of Atoh1a, DeltaA or etv4 helps correlate the periodic pattern of Fgf signaling with periodic morphogenesis of rosettes and deposition of neuromasts. However, it is not clear what the morphogenesis of poorly formed rosettes within the migrating primordium has to do with the ultimate pattern of deposited neuromasts in mcf2lb mutants. Both sets of videos suggest that primordium cells seem to lose their organization as rosettes

© 2024. Published by The Company of Biologists under the terms of the Creative Commons Attribution License (https://creativecommons.org/licenses/by/4.0/).

while depositing, they deposit essentially unclustered, and they seem to re-aggregate post-deposition to re-form rosettes of variable size, though this is hard to see in the higher mag videos that were designed to focus on morphogenesis within the primordium. The authors should address this issue and determine if there indeed a disconnect between the cells that initially aggregate to form poorly formed rosettes in the primordium and how they ultimately reorganize following deposition? If there is a disconnect, what accounts for the patten of aggregation of deposited cells?

- The Reviewer is correct, as we also noticed the deposition phenotype during our analysis of *mcf2lb* mutants. However, this observation did not make it into the initial manuscript, as we focused on the role of Mcf2lb within the pLLP. It is possible that cell-cell adhesion is affected in *mcf2lb* mutants specifically during deposition. Interestingly, this more unclustered and fragmented rosette appearance is not observed while the cells are in the migrating pLLP. Instead, it only appears while these cells are being deposited and then ultimately resolves as the pLLP continues to migrate. Future studies are needed to examine cell adhesion both within the migrating pLLP and during deposition in *mcf2lb* mutants. We added additional sentences to address this point in the discussion on Page 22 and 23, Lines 678-682.

In summary, we believe we adequately addressed reviewer 2 and 3’s comments in the revised version of our manuscript. Please do not hesitate to let us know if we can provide any more information in support of our work for publication in *Development*.

Third decision letter

MS ID#: DEVELOP/2023/201898

MS TITLE: RhoA GEF Mcf2lb regulates rosette integrity during collective cell migration

AUTHORS: Hannah M Olson, Amanda Maxfield, Nicholas L Calistri, Laura M Heiser, Weiyi Qian, Holger Knaut, and Alex V Nechiporuk
ARTICLE TYPE: Research Article

I have looked carefully at your response to the remaining comments of reviewers and in light of this I am happy to tell you that your manuscript has been accepted for publication in Development, pending our standard ethics checks.

© 2024. Published by The Company of Biologists under the terms of the Creative Commons Attribution License (https://creativecommons.org/licenses/by/4.0/).
